# Targeting intrinsically disordered nuclear protein 1 (NUPR1) with single-domain antibodies alleviates triple-negative breast cancer (TNBC) progression in vivo

**DOI:** 10.1038/s41419-025-08332-2

**Published:** 2025-12-22

**Authors:** Tianzhuo Wang, Min Wang, Xuanru Chen, Yueyuan Yin, Jintao Xu, Yanan Sun, Ailing Wu, Zhe Liu, Zhenyi Ma

**Affiliations:** 1 Zhejiang Key Laboratory of Medical Epigenetics, Department of Biochemistry and Molecular Biology, School of Basic Medical Sciences, Hangzhou Normal University, Hangzhou, Zhejiang China; 2 Department of Clinical Laboratory, The Second Qilu Hospital of Shandong University, Jinan, Shandong China; 3 Tianjin Key Laboratory of Medical Epigenetics, Key Laboratory of Immune Microenvironment and Disease (Ministry of Education), Department of Immunology, Biochemistry and Molecular Biology, School of Basic Medical Sciences, Tianjin Medical University, Tianjin, China

**Keywords:** Breast cancer, Antibody fragment therapy

## Abstract

Triple-negative breast cancer (TNBC) is a highly aggressive subtype that currently lacks effective targeted therapies. Transcriptional co-regulator nuclear protein 1 (NUPR1) has been identified as a key stress-adaptive disordered protein that promotes tumor progression and therapy-induced resistance. In this study, we developed a robust high-throughput platform integrating in situ proximity ligation assay followed by DNA sequencing (*is*PLA-seq), NanoBiT assays, and C-degron degradation validation to screen for functional single-domain antibodies (sdAbs) targeting NUPR1. Consequently, sdAb#07.81 emerged as a lead candidate, demonstrating strong binding affinity and the ability to degrade endogenous NUPR1 in TNBC cells. Functional assays confirmed that sdAb#07.81 suppressed TNBC cell growth, induced premature senescence, and derepressed ferroptosis. In vivo validation using a 4T1 cell-derived fully immunocompetent murine model further established its therapeutic efficacy, with significant reductions in tumor size, NUPR1 expression, and cell proliferation. These findings highlight sdAb#07.81 as a promising therapeutic agent and validate the platform’s effectiveness for addressing intracellular disordered targets like NUPR1. This work underscores the potential of sdAbs as a cancer therapeutic and provides a foundation for advancing sdAb#07.81 into preclinical and clinical development to address the critical unmet needs of TNBC treatment.

## Introduction

Triple-negative breast cancer (TNBC) represents one of the most aggressive subtypes of breast cancer, accounting for ~15–20% of all breast cancer cases [1, 2]. Unlike hormone receptor-positive and HER2-positive breast cancers, TNBC lacks effective targeted therapies; chemotherapy remains the standard of care [3, 4]. Among them, a key hallmark of TNBC is its reliance on stress response mechanisms to survive the hostile tumor microenvironments [5]. As a stress-inducible disordered protein, nuclear protein 1 (NUPR1, also known as p8 or Com-1), regulates diverse processes [6–9], including chromatin remodeling [10], transcriptional activation [11], autophagy [12], premature senescence [13], ferroptosis [14, 15], and adaptation to stress [15, 16]. Among them, we had successively discovered the crucial roles of the autophagy-lysosome and premature senescence pathway regulated by NUPR1 in non-small cell lung cancer and the drug resistance of ER^+^ breast cancer [13, 17]. Importantly, the elevated NUPR1 expression has been implicated in tumor progression, therapy-induced resistance, and poor clinical outcomes in pancreatic cancer [18, 19], oral squamous cell carcinoma [12, 20], lung cancer [17, 21], liver cancer [22, 23], renal cancer [24], and breast cancer [25, 26], emerging as a promising therapeutic target.

Single-domain antibody (sdAb, also called nanobody) has emerged as a novel therapeutic modality with unique advantages [27]. Derived from the variable domains of camelid heavy-chain-only antibodies, sdAbs are characterized by their small size, high specificity, and exceptional stability [28–30]. Unlike conventional monoclonal antibodies, sdAbs can penetrate tumors more effectively and target cryptic or intracellular epitopes [31], making them particularly suited for addressing challenging targets such as NUPR1. Additionally, their simple structure facilitates genetic engineering and large-scale production, enhancing their potential for clinical application [32, 33]. However, developing functional sdAbs requires robust, high-throughput screening strategies that ensure both specificity and functionality.

This study presents a robust, high-throughput screening platform based on in situ proximity ligation assay followed by DNA sequencing (*is*PLA-seq) [34]-NanoBiT assay-C-degron degradation validation for discovering functional sdAbs targeting NUPR1 and provides compelling evidence for their therapeutic potential in TNBC. By integrating with rigorous functional validation, we establish a foundation for the clinical development of anti-NUPR1 sdAb#07.81. Thus, this work establishes the therapeutic rationale for targeting the disordered NUPR1 intracellularly with the identified sdAbs, providing a robust way for further investigations to translate our findings into viable TNBC therapies.

## Results

### Strategy for screening sdAbs against NUPR1

**Fig. 1:**
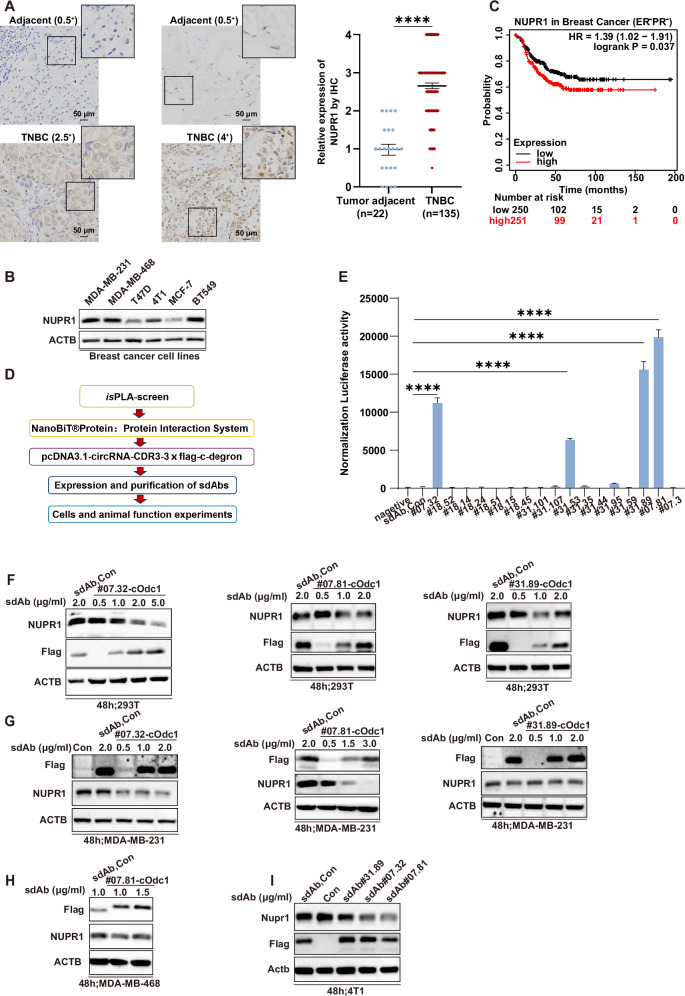
High-sensitive, high-throughput screening for anti-NUPR1 sdAbs. **A** Representative distribution of NUPR1 by IHC in clinical triple-negative breast cancer specimens and adjacent noncancerous tissues from the patients of origin (IHC, brown). Representative images were shown. Scale bars: 50 μm (left). Semiquantitative analysis and unpaired Student’s *t* test were performed to compare the levels of NUPR1 between human clinical triple-negative breast cancer specimens and adjacent noncancerous tissues. Data are presented as the mean ± SEM, ****P < 0.0001 (right). **B** NUPR1 levels were examined in the indicated breast cancer cell lines by immunoblots. **C** NUPR1 survival curves in patients with ER and PR negative breast cancer were calculated using the K-M plot analysis. **D** Workflow of anti-NUPR1 sdAbs screening. First, CDR3 was screened by *is*PLA assay. Second, using *is*PLA screen positive CDR3 sequences verified by NanoBiT assay. Third, using NanoBiT positive CDR3 sequences verified by C-degron assay, and then purification and functional validation of anti-NUPR1 sdAbs. **E** *is*PLA screen positive NUPR1 CDR3 sequences verified by NanoBiT assay. Luciferase reporter activity of each construct co-transfected with vector, LgBiT-CDR3, and SmBiT-NUPR1 in HEK293 cells. **F**–**I** Using *is*PLA and NanoBiT positive CDR3 sequences verified by C-degron assay. The results showed that sdAb#07.32 and sdAb#07.81 had a better degradation effect on NUPR1. **F** Both *is*PLA screen and NanoBiT positive CDR3 sequences verified by C-degron (cOdc1) assay by immunoblot. HEK293T cells were transfected with increasing amounts of Flag-sdAb#07.32-cOdc1 (left), or Flag-sdAb#07.81-cOdc1 (middle), or Flag- sdAb#31.89-cOdc1 (right) expression plasmid, compared with Flag-sdAb-Con, and endogenous protein levels of NUPR1 were determined. ACTB acts as a loading control. **G** MDA-MB-231 cells were transfected with increasing amounts of Flag-sdAb#07.32-cOdc1 (left), or Flag-sdAb#07.81-cOdc1 (middle), or Flag-sdAb #31.89-cOdc1 (right) expression plasmid, compared with Con or Flag-sdAb-Con, and endogenous protein levels of NUPR1 were determined. ACTB acts as a loading control. **H** MDA-MB-468 cells were transfected with increasing amounts of Flag-sdAb#07.81-cOdc1 expression plasmid, compared with Flag-sdAb-Con, and endogenous protein levels of NUPR1 were determined. ACTB acts as a loading control. **I** 4T1 cells were transfected with Flag-sdAb#07.81-cOdc1, or Flag-sdAb#07.32-cOdc1, or Flag-sdAb#31.89-cOdc1 expression plasmid, compared with Con and Flag-sdAb-Con, and endogenous protein levels of Nupr1 were determined. Actb acts as a loading control.

To confirm the upregulation of NUPR1 in TNBC pathological tissue, we first performed immunohistochemistry (IHC) analyses using human TNBC tissue microarrays. Compared with the tumor adjacent, NUPR1 expression was higher in TNBC (Fig. 1A). This was consistent with high protein levels of NUPR1 in TNBC cell lines (MDA-MB-231, MDA-MB-468, 4T1 and BT549 cells are TNBC cell lines; T47D and MCF-7 are ER positive breast cancer cell lines.) (Fig. 1B). Kaplan–Meier analysis showed that high NUPR1 expression was also strongly associated with a poor prognosis in ER and PR negative breast cancer patients (Fig. 1C). Given the critical role of NUPR1 in breast cancer, we next developed a high-throughput screening platform for sdAbs targeting NUPR1, aiming to provide a new therapeutic approach for TNBC (Fig. 1D). Using this sdAb screening approach named *is*PLA-seq, we screened a synthesized C-terminal Flag-tagged sdAb library against HA-tagged NUPR1 and 17 sdAbs were enriched with their CDR3 amino acid sequences after three rounds of screening as listed in Table 1. Furthermore, based on the NanoBiT assay results, sdAb#07.32, sdAb#07.81, and sdAb#31.89 demonstrated greater effectiveness, exhibiting strong luciferase activity indicative of specific binding to NUPR1 in living cells. Notably, sdAb#07.81 showed the strongest activity (Fig. 1E). Functional validation using the C-terminus of ornithine decarboxylase (cOdc1, a native C-degron) [35] degradation assay across HEK293T, MDA-MB-231, MDA-MB-468, and 4T1 cells confirmed the ability of these sequences to induce NUPR1 degradation. Both Flag-sdAb#07.32-cOdc1 and Flag-sdAb#07.81-cOdc1 demonstrated dose-dependent reductions in endogenous NUPR1 protein levels, with sdAb#07.81 showing superior activity across all these cell lines, except for Flag-sdAb#31.89-cOdc1 (Fig. 1F–I). These findings validate the effectiveness of the high-throughput screening platform and establish sdAb#07.81 as a potent candidate for further development as a therapeutic sdAb for TNBC.

**Table 1:** The CDR3 sequences of anti-NUPR1 sdAb candidates.

#	Amino acid sequence	Round 2/3
#18.45	EFSRSRIFGGVQEPCGLQGRI	1400/32
#18.15	GRELRANWLLARQPAEPEMFC	1299/31
#07.32	IVTGLNCRFVAGSRGVSYHKA	1298/1590
#07.81	LSGPSWDWECLSAIVASGELN	1224/2222
#18.14	PSLFGQRRNFRAVVVPRLFDW	485/12
#07.3	PGGGSAYLTMGIRYVHWYCSH	424/489
#18.51	GGTVLRFVGMLLEQRCFGSGG	334/9
#31.35	RRITVVSLADRRHYVSLEGHE	126/8
##31.44	VVGLLASLRLTAVAACVGLLH	103/10
##31.53	SSKGVYVSVGCNGGRADLLFH	82/6
##31.59	DMGKRYLLCVEVPCNDNLPRR	74/8
##18.52	PTGGRCGFGGEEVSNDVTRRV	49/1
##31.89	ILHESDCRTSGLVVVTELIRA	37/3
##31.95	GTYYPVVNTEIRVESISLFEA	35/6
##18.24	GLGSWPFPIFAGGSRSYVVYG	36/487
##31.101	PKRRTFGLEWFRTCGSPGHLL	32/5
##31.107	EFGNDVQSVAFKYVVGRGYQL	26/65

### Live-cell imaging and functionality of positive NUPR1 sdAbs

**Fig. 2:**
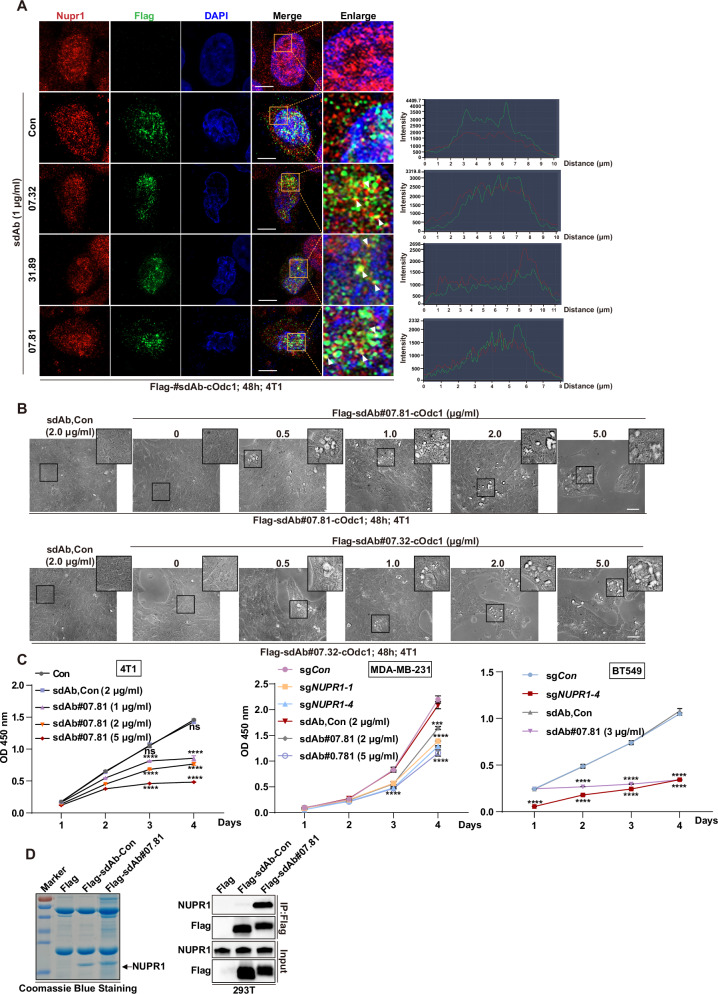
Live-cell imaging and functionality of positive NUPR1 sdAbs. **A** 4T1 cells were transfected with Flag-sdAb#07.81-cOdc1, or Flag-sdAb#07.32-cOdc1, or Flag-sdAb#31.89-cOdc1 expression plasmid, compared with Con and Flag-sdAb-Con. 4T1 cells were subjected to triple immunofluorescence staining (IF) with anti-Flag mouse (green), anti-NUPR1 rabbit (red), and DAPI (blue), followed by visualization with confocal microscopy. White arrows showed the yellow points and intensity curve on the right referring co-localization condition. Scale bars: 10 μm. **B** Cell images of 4T1 cells were transfected with increasing amounts of Flag-sdAb#07.81-cOdc1 expression plasmid and Flag-sdAb#07.32-cOdc1 expression plasmid, compared with Flag-sdAb-Con. Scale bars, 50 μm. **C** Cell growth of 4T1 cells (left), MDA-MB-231 cells (middle), and BT549 cells (right) were measured by CCK-8 assay. Data are presented as the mean ± SEM of three independent experiments; the error bars show one standard deviation. ***P < 0.001, ****P < 0.0001. **D** HEK293T cells were transfected with Flag, Flag-sdAb-Con or Flag-sdAb#07.81 and used for IP and then subjected to Coomassie brilliant blue staining (left), and followed by immunoblot with NUPR1 and Flag antibodies (right).

The functional verification of positive NUPR1-specific sdAbs in this part involved a series of experimental techniques to assess the expression, localization, and functional impact of the sdAb C-degron (cOdc1) [35] plasmids on cells. Initially, 4T1 cells, TNBC cell line from mice, were transfected with various Flag-tagged expression plasmids, including Flag-sdAb#07.81-cOdc1, Flag-sdAb#07.32-cOdc1, and Flag-sdAb#31.89-cOdc1, in comparison with control plasmids Flag-sdAb-Con and Con. These transfected cells underwent triple immunofluorescence (IF) staining, using anti-Flag mouse antibody (green), anti-NUPR1 rabbit antibody (red), and DAPI (blue) to identify cellular co-localization. Confocal microscopy images revealed yellow punctate regions and quantifiable intensity curves supporting these observations, indicating that NUPR1 and Flag-sdAb#07.81-cOdc1, Flag-sdAb#07.32-cOdc1 co-localized in 4T1 cells (Fig. 2A), especially Flag-sdAb#07.81-cOdc1. However, the co-localization of NUPR1 and Flag-sdAb#31.89-cOdc1 is not ideal (Fig. 2A). In addition, cell images taken under increasing plasmid amounts further demonstrated a dose-dependent cytoplasmic vacuolization of the Flag-sdAb#07.81-cOdc1 and Flag-sdAb#07.32-cOdc1 plasmids compared to the Flag-sdAb-Con (Fig. 2B). The study further evaluated the impact of Flag-sdAb#07.81-cOdc1 plasmids on cell proliferation in 4T1, MDA-MB-231, and BT549 cell lines using a CCK-8 assay and compared it with stable knockout NUPR1 cell lines. The results showed a significant reduction in cell growth when transfected with increasing amounts of Flag-sdAb#07.81-cOdc1 plasmid (*P* < 0.0001) compared to Con or Flag-sdAb-Con-cOdc1. These effects caused by these treatments were consistent with *NUPR1* depletion (Fig. 2C and Supplementary Fig. 1A, B). Finally, this part performed immunoprecipitation (IP) experiments in HEK-293T cells transfected with Flag, Flag-sdAb-Con, or Flag-sdAb#07.81 plasmids. After Coomassie brilliant blue staining and immunoblotting with NUPR1 and Flag antibodies, the results showed that endogenous NUPR1 and Flag-sdAb#07.81 interacted in HEK293T cells (Fig. 2D). These experiments collectively support the potential of specific NUPR1-targeted sdAb#07.81 as a promising tool for functional analysis and therapeutic applications.

### Functional validation of Flag-tagged anti-NUPR1 sdAbs in vitro and in vivo

**Fig. 3:**
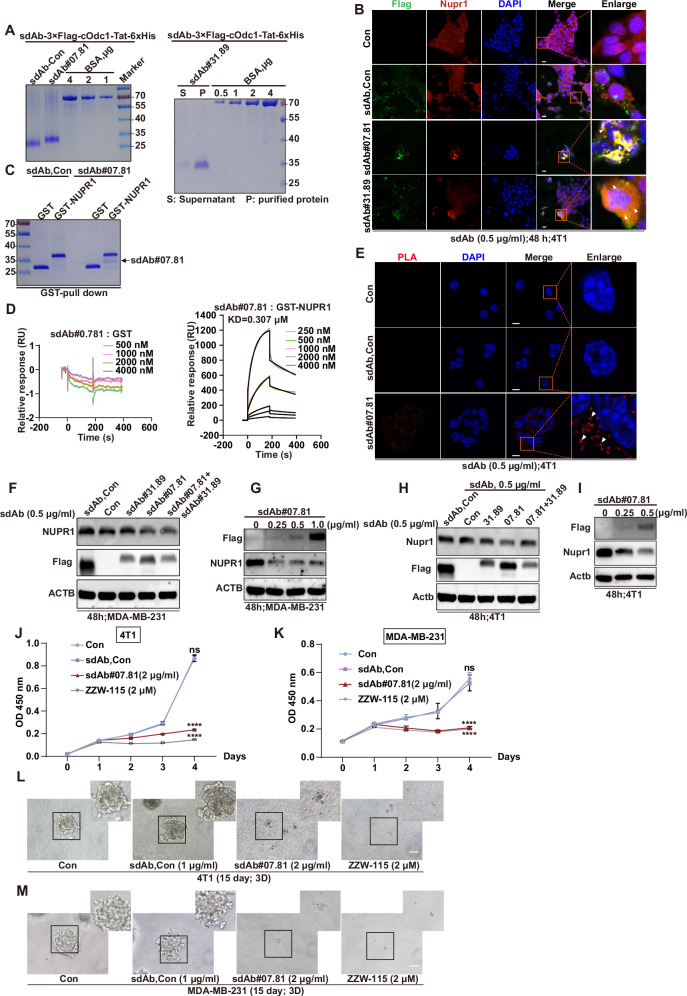
Functional validation of Flag-tagged anti-NUPR1 sdAbs in vitro. **A** The purification of sdAb-Con-3xFlag-cOdc1-Tat-6xHis (left), sdAb#07.81-3xFlag-cOdc1-Tat-6xHis (left), and sdAb#31.89-3xFlag-cOdc1-Tat-6xHis (right) from HEK293F followed by Coomassie blue staining with BSA as a loading control. S supernatant, P purified sdAb protein. **B** 4T1 cells were subjected to triple IF with anti-Flag mouse (green), anti-NUPR1 rabbit (red), and DAPI (blue), followed by visualization with confocal microscopy. White arrows showed the yellow points referring to co-localization. Purified sdAb-Con, sdAb#07.81, and sdAb#31.89 proteins were added to 4T1 cells, and IF was performed after 48 h. Scale bars: 20 μm. **C** GST pull-down assays were performed using purified sdAb-Con or sdAb#07.81, and then incubated with purified GST or GST-NUPR1 at 4 °C overnight. The remaining proteins were resolved by SDS-PAGE and further analyzed by Coomassie blue staining. **D** SPR sensorgram between sdAb-Con: GST (left); SPR sensorgram between sdAb#07.81: GST-NUPR1 (right). **E** Functional validation of anti-NUPR1 sdAb #07.81 by in *is*PLA using anti-NUPR1 and anti-Flag antibodies at 48 h after transfection, as indicated by white arrow heads. Nuclei are counterstained with DAPI (blue). Scale bar, 10 μm. **F**–**I** Immunoblot of NUPR1 and Flag in MDA-MB-231cells (**F**, **G**) or 4T1 (**H**, **I**) treated with purified anti-NUPR1 sdAb clone Con, #31.89, #07.81, and #31.89 + #07.81 (0.5 μg/mL) at the indicated concentration for 48 h, compared with Con. ACTB was used as a loading control. **J**, **K** Cell growth of 4T1 cells (**J**), MDA-MB-231 cells (**K**) was measured by CCK-8 assay. Data are presented as the mean ± SEM of three independent experiments; the error bars show one standard deviation. ****P < 0.0001. **L**, **M** 4T1 (**L**) or MDA-MB-231 cells (**M**) treated with purified anti-NUPR1 sdAb clone Con, #07.81(2 μg/mL) or ZZW-115 (2 μM) were cultured in Matrigel for 15 days. Scale bars: 100 μm. Three independent experiments were performed. Data shown as mean ± SEM. ****P < 0.0001.

In this section, the functional validation of Flag-tagged anti-NUPR1 sdAbs were conducted in vitro through a rigorous and systematic experimental approach. First, sdAb-Con-3xFlag-cOdc1-Tat-6xHis (left), sdAb#07.81-3xFlag-cOdc1-Tat-6xHis (left), and sdAb#31.89-3xFlag-cOdc1-Tat-6xHis (right) were successfully purified from HEK293F cells and analyzed using Coomassie blue staining (Fig. 3A). Next, 4T1 cells underwent triple IF staining using anti-Flag mouse antibody (green), anti-NUPR1 rabbit antibody (red), and DAPI (blue). Confocal microscopy images revealed yellow punctate regions supporting these observations, indicating that NUPR1 and sdAb#07.81, sdAb#31.89 co-localized in 4T1 cells (Fig. 3B), especially sdAb#07.81. For the positive sdAbs against NUPR1, a glutathione-S-transferase (GST) pulldown assay between GST-tagged NUPR1 truncations and sdAbs further confirmed the direct interaction of sdAb#07.81 with NUPR1, compared with sdAb-Con (Fig. 3C). Surface plasmon resonance (SPR) assay could also demonstrate the direct interaction between GST-NUPR1 and sdAb#07.81 in vitro (Fig. 3D). Further, for validation of their binding activity in vivo, sdAb#07.81 or sdAb-Con was added into 4T1 cells, followed by *is*PLA. As shown in the *is*PLA signals, the anti-NUPR1 sdAb#07.81 interacted with the endogenous NUPR1 compared to the negative sdAb-Con, indicating that sdAb#07.81 could not only enter 4T1 cells, but also bind to endogenous NUPR1 (Fig. 3E). Functional validation using the C-degron (cOdc1) [35] degradation assay across MDA-MB-231 and 4T1 cells confirmed the ability of sdAb proteins to induce NUPR1 degradation. The sdAb#07.81 (0.5 μg/ml) demonstrated a reduction in endogenous NUPR1 protein levels compared to Con and sdAb, besides sdAb#31.89 (Fig. 3F–I).

To further comprehensively evaluate the therapeutic efficacy of the sdAb#07.81 in TNBC, we performed a pharmacological comparative analysis in comparison to a potent NUPR1 inhibitor ZZW-115 [36–38]. Specifically, CCK-8 assays (Fig. 3J, K), 2D (Supplementary Fig. 2A, B), and 3D culture assays (Fig. 3L, M) consistently revealed that both the sdAb#07.81 and ZZW-115 suppressed the proliferation and spheroid-forming capacity of TNBC cells, with similar efficacy between these two treatments. In this part, we found that our designed sdAb#07.81 could not only directly bind to NUPR1 but also enter cells to exert its inhibitory functions against breast cancer cells. Meanwhile, it laid the foundation for the effectiveness of subsequent animal experiments.

### Anti-NUPR1 sdAb#07.81 suppresses the progression of TNBC cells in vivo

**Fig. 4:**
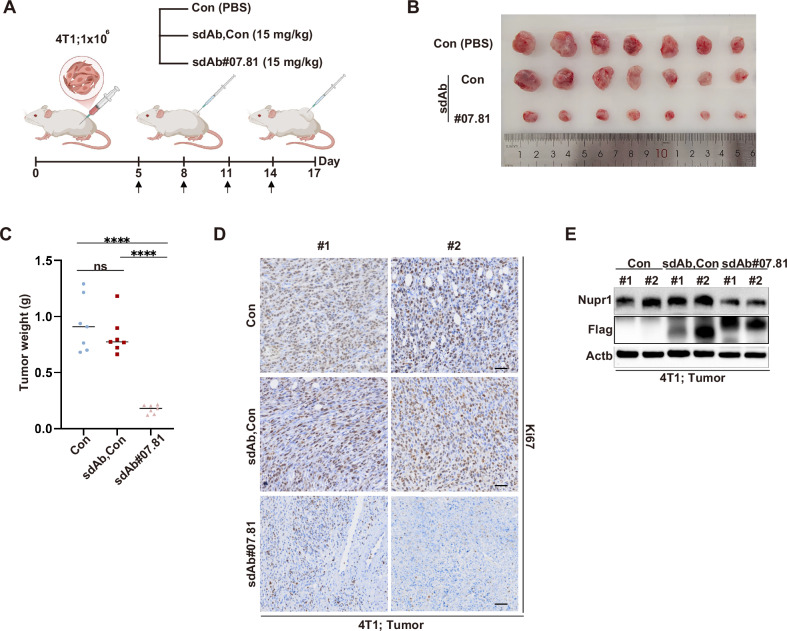
Anti-NUPR1 sdAb#07.81 inhibits 4T1 cell progression in vivo. **A** Allograft experiment was performed on BALB/c mice injected with 4T1 cells (1 × 10^6^ cells per mouse). Groups of different mice that were Con (*n* = 7), sdAb-Con (*n* = 7) or sdAb#07.81 (*n* = 7). Tumor was injected every 3 days with purified sdAb proteins. **B** Representative images of tumors from (**A**). **C** Tumors weight was measured. ****P < 0.0001 by unpaired Student’s *t* test. Data are presented as the mean ± SEM and are from one independent experiment with seven mice per group. **D** Representative images of IHC staining of Ki67 in subcutaneous tumor tissue sections. Scale bars: 200 μm. **E** The levels of Flag and Nupr1 in breast tissues were measured using immunoblot. Actb was used as a loading control.

To explore the role played by anti-NUPR1 sdAb#07.81 in TNBC progression, 4T1 cells were subcutaneously injected into BALB/c mice (1 × 10^6^ cells per mouse). The mice were divided into three groups: Con (PBS), sdAb-Con and sdAb#07.81, each consisting of seven mice. Purified sdAb proteins with 15 mg/kg were injected into the tumors every three days (Fig. 4A). Representative images of tumors from each group showed a noticeable difference in tumor size (Fig. 4B), supported by a significant reduction in tumor weight in the sdAb#07.81 group compared to the Con group and sdAb-Con group (Fig. 4C). Further, tumor tissues stained for Ki67 via immunohistochemistry demonstrated reduced proliferation in the sdAb #07.81 group, with representative images displaying distinct differences (Fig. 4D). In addition, immunoblot analysis of 4T1 tumors indicated decreased levels of NUPR1 proteins in the sdAb#07.81-treated group compared to the Con group and sdAb-Con group (Fig. 4E). To assess the in vivo toxicity profile of this sdAb#07.81, we performed intratumoral administration at supra-therapeutic doses. We measured the body weight and survival rate of mice in each group and performed necropsy on major organs (heart, liver, and kidney) (Supplementary Fig. 3A–D). Notably, we did not find any appreciable adverse toxic effects compared with Con and sdAb#Con groups, including in a 16-day treatment period using 30 mg/kg. Conversely, the sdAb#07.81 group showed a significant increase in body weight relative to the Con group and the sdAb-Con group, potentially attributable to effective tumor suppression. Together, these findings demonstrated that the identified sdAb#07.81 not only suppressed TNBC progression in vivo but also possessed a reasonable safety profile.

### Anti-NUPR1 sdAb#07.81 suppress the progression of TNBC cells via induces premature senescence and derepresses ferroptosis

**Fig. 5:**
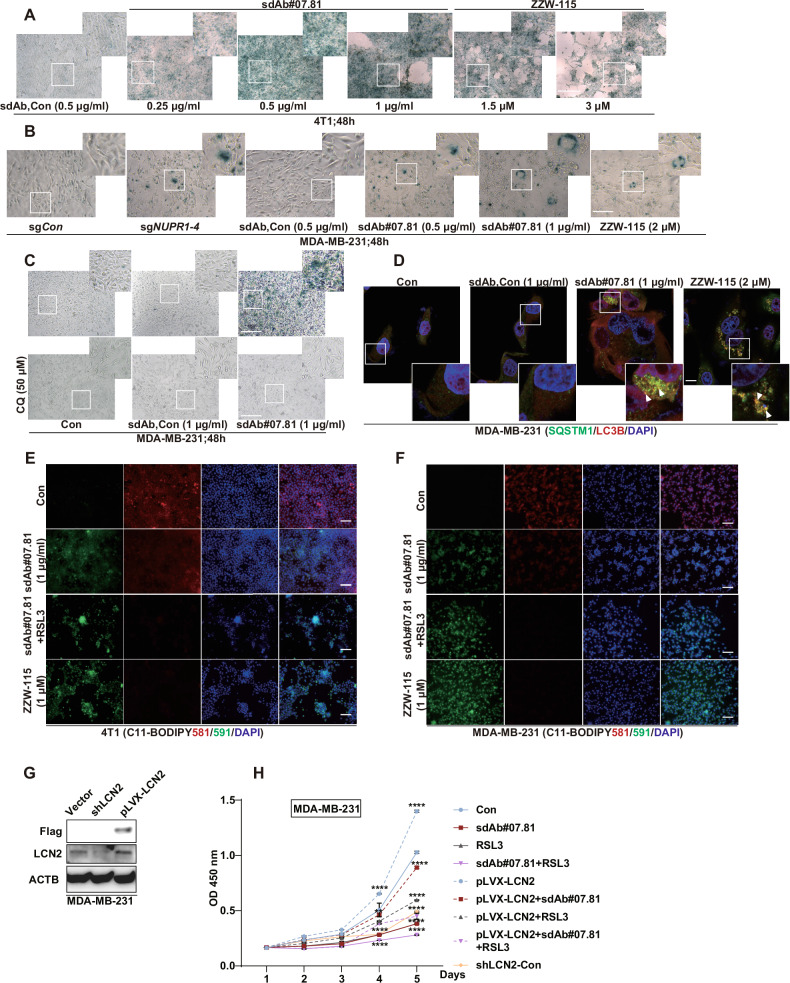
Anti-NUPR1 sdAb#07.81 induces premature senescence and derepresses ferroptosis. **A** Representative light microscopy images of GLB1 staining in 4T1 added with purified sdAb-Con, anti-NUPR1 sdAb#07.81 protein or NUPR1 inhibitor ZZW-115 at the indicated concentration for 48 h (*n* = 3), Scale bar, 200 μm. **B** Representative light microscopy images of GLB1 staining in MDA-MB-231 (Con) or sg*NUPR1*, or MDA-MB-231 added with purified sdAb-Con, anti-NUPR1 sdAb#07.81 protein or ZZW-115 at the indicated concentration for 48 h (*n* = 3), Scale bar, 200 μm. **C** Representative light microscopy images of GLB1 staining in MDA-MB-231 treated with chloroquine (CQ) or not, and added with purified sdAb-Con or anti-NUPR1 sdAb#07.81 protein for 48 h (*n* = 3), Scale bar, 200 μm. **D** MDA-MB-231 cells were subjected to triple IF with anti-SQSTM1 mouse (green), anti-LC3B rabbit (red), and DAPI (blue), followed by visualization with confocal microscopy. White arrows showed the yellow points referring to co-localization. Purified sdAb-Con, sdAb#07.81 proteins or ZZW-115 were added to MDA-MB-231 cells, and IF was performed after 48 h. Scale bars: 100 μm. **E**, **F** Lipid peroxidation measurement via C11-BODIPY live-cell confocal imaging in 4T1 and MDA-MB-231 cells following 24 h treatment with anti-NUPR1 sdAb#07.81, RSL3, and sdAb#07.81 or ZZW-115. Three independent experiments were performed. **G** Establishment of stable LCN2 overexpression and knockdown MDA-MB-231 cell Lines. The levels of Flag and LCN2 were measured using an immunoblot. ACTB used as a loading control. **H** Cell growth of MDA-MB-231 cells was measured by CCK-8 assay. Data are presented as the mean ± SEM of three independent experiments; the error bars show one standard deviation. ****P < 0.0001.

Previous work has indicated that the loss of NUPR1 suppresses tumor progression by inducing premature senescence [13] and ferroptosis in breast cancer [25]. To investigate this, we first performed GLB1 staining (a marker of premature senescence) on 4T1 and MDA-MB-231 cells treated with sdAb-Con or sdAb#07.81 at varying concentrations. Representative light microscopy images are shown. We found that GLB1 staining gradually deepened with the increased amount of sdAb#07.81, indicating that the number of premature senescent cells was also gradually increased (Fig. 5A, B). In contrast, chloroquine (CQ, autophagy inhibitor) treatment partially rescued staining for GLB1 (Fig. 5C). The IF results demonstrated that the addition of the sdAb#07.81 proteins led to upregulated SQSTM1 (green) protein expression, increased co-localization with LC3B (red), and the formation of LC3B puncta (Fig. 5D).

To detect the activation of ferroptosis by sdAb#07.81, lipid peroxidation was assessed via C11-BODIPY staining and live-cell fluorescence imaging. Treatment with sdAb#07.81 alone significantly induced lipid peroxidation, which was further augmented by co-treatment with the established ferroptosis activator RSL3 (Fig. 5E, F). These results are consistent with corresponding sg*NUPR1* or ZZW-115 in TNBC cells (Fig. 5A–F). NUPR1 can protect cancer cells from excess iron by inducing the expression of the iron-sequestering protein lipocalin-2 (LCN2) [15, 39]. According to the CCK-8 assay, adding sdAb#07.81 significantly inhibited the cell proliferation promoted by LCN2 overexpression, with an effect similar to that of LCN2 knockdown. Strikingly, a synergistic inhibition was observed when sdAb#07.81 was combined with RSL3 (Fig. 5G, H). Collectively, these findings indicated that sdAb#07.81 inhibits tumor progression by concurrently promoting premature senescence and the ferroptosis pathway.

**Fig. 6:**
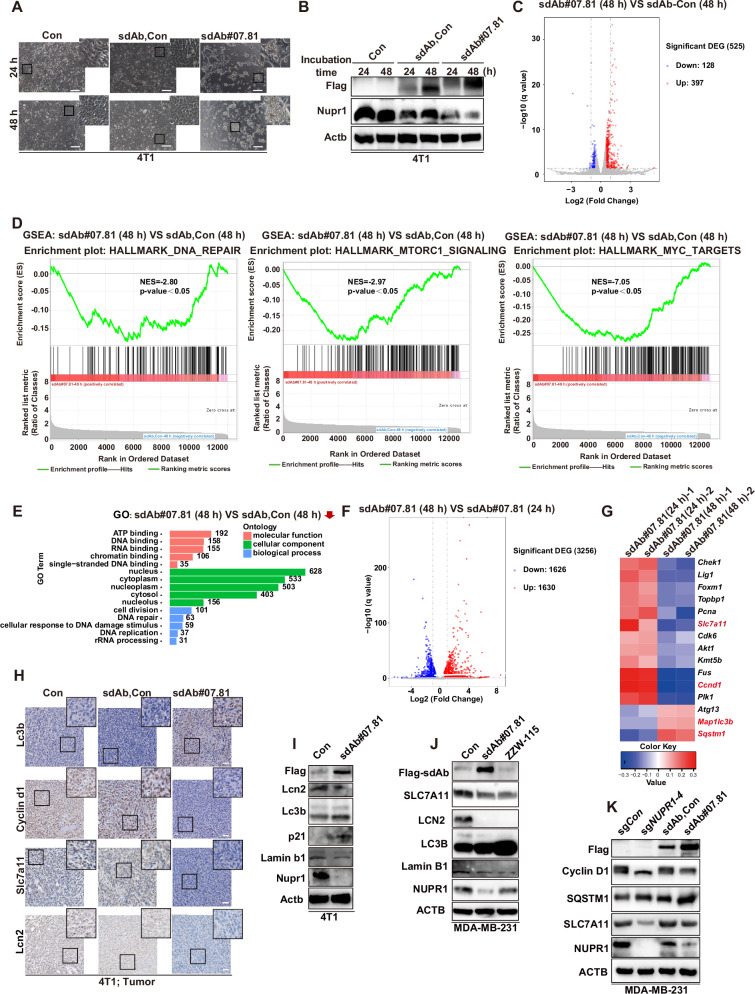
Anti-NUPR1 sdAb#07.81 downregulates cancer progression-related pathways. **A**, **B** Cell images of 4T1 cells were treated with purified anti-NUPR1 sdAb clone Con, sdAb#07.81 (0.5 μg/mL) for 24 h or 48 h, compared with Con (**A**). Scale bars, 200 μm. The levels of Flag and Nupr1 in 4T1 cells were measured using immunoblot. Actb was used as a loading control (**B**). **C**–**G** 4T1 cells from Fig. 5A treatment were measured using RNA-Seq. **C** The volcano plot was shown in sdAb#07.81 (48 h) versus sdAb-Con (48 h). **D** GSEA enrichment analysis showed that DNA repair, mTORC1 signaling and MYC targets related pathways were downregulated in sdAb#07.81 (48 h) versus sdAb-Con (48 h). **E** GO enrichment analysis showing the top 15 downregulated pathways in sdAb#07.81 (48 h) versus sdAb#07.81 (24 h). **F** The volcano plot was shown in sdAb#07.81 (48 h) versus sdAb#07.81 (24 h). **G** Functional profiling of genes differentially expressed between sdAb#07.81 (48 h) and sdAb#07.81 (24 h) in cell proliferation, autophagy, and ferroptosis-related pathways. Representative upregulated (red) and downregulated genes (blue) upon sdAb#07.81 are listed vertically (right). **H** Representative images of IHC staining of Lc3b, Cyclin d1, Slc7a11, and lcn2 in subcutaneous tumor tissue sections. Scale bars: 200 μm. **I** Immunoblot of Flag, Lcn2, Lc3b, p21, Lamin b1, Nupr1 in 4T1 cells treated with sdAb#07.81(1 μg/mL) for 48 h, compared with Con. Actb was used as a loading control. **J** Immunoblot of Flag, SLC7A11, LCN2, LC3B, Lamin B1 and NUPR1 in MDA-MB-231cells treated with sdAb#07.81(1 μg/mL) or ZZW-115 (2 μM) for 48 h, compared with Con. ACTB was used as a loading control. **K** Immunoblot of Cyclin D1, SLC7A11, SQSTM1, NUPR1 and Flag in MDA-MB-231cells treated with sg*NUPR1*, anti-NUPR1 sdAb-Con and sdAb#07.81(0.5 μg/mL) for 48 h, compared with Con. ACTB was used as a loading control.

To gain deeper insights into the mechanism underlying the action of sdAb#07.81 in inhibiting TNBC progression, we treated with purified anti-NUPR1 sdAb clone Con, #07.81 (0.5 μg/mL) for 24 h or 48 h, compared with Con (Fig. 6A, B). 4T1 cells from Fig. 6A treatment were tested the transcriptomic change by RNA-Seq. The volcano plot was shown in sdAb#07.81 (48 h) versus sdAb-Con (48 h) (Fig. 6C). We found that DNA repair, mTORC1 signaling, MYC targets, DNA binding, RNA binding, DNA repair and DNA replication-related pathways were downregulated in sdAb#07.81 (48 h) versus sdAb-Con (48 h) from GSEA enrichment analysis (Fig. 6D) and GO enrichment analysis (Fig. 6E). These results are consistent with previous research of shNUPR1 [6, 13, 40]. Further, the volcano plot and heatmap analysis also showed that compared to the sdAb#07.81 (24 h) treatment group, the sdAb#07.81 (48 h) treatment group showed further upregulation in premature senescence and ferroptosis-related pathways (Fig. 6F, G). Similar to genetic *NUPR1* depletion [13, 17, 37] or ZZW-115 treatment [15, 38], pharmacological NUPR1 targeting by sdAb#07.81 consistently induced a concerted biological response in TNBC models both in vitro and in vivo. This response was characterized by the progressive activation of a premature senescence program (SQSTM1, LC3B II, p21) concurrent with the suppression of key survival proteins involved in ferroptosis defense (SLC7A11, LCN2) and senescence evasion (Cyclin D1 and Lamin B1) (Fig. 6H–K and Supplementary Fig. 3E).

**Fig. 7:**
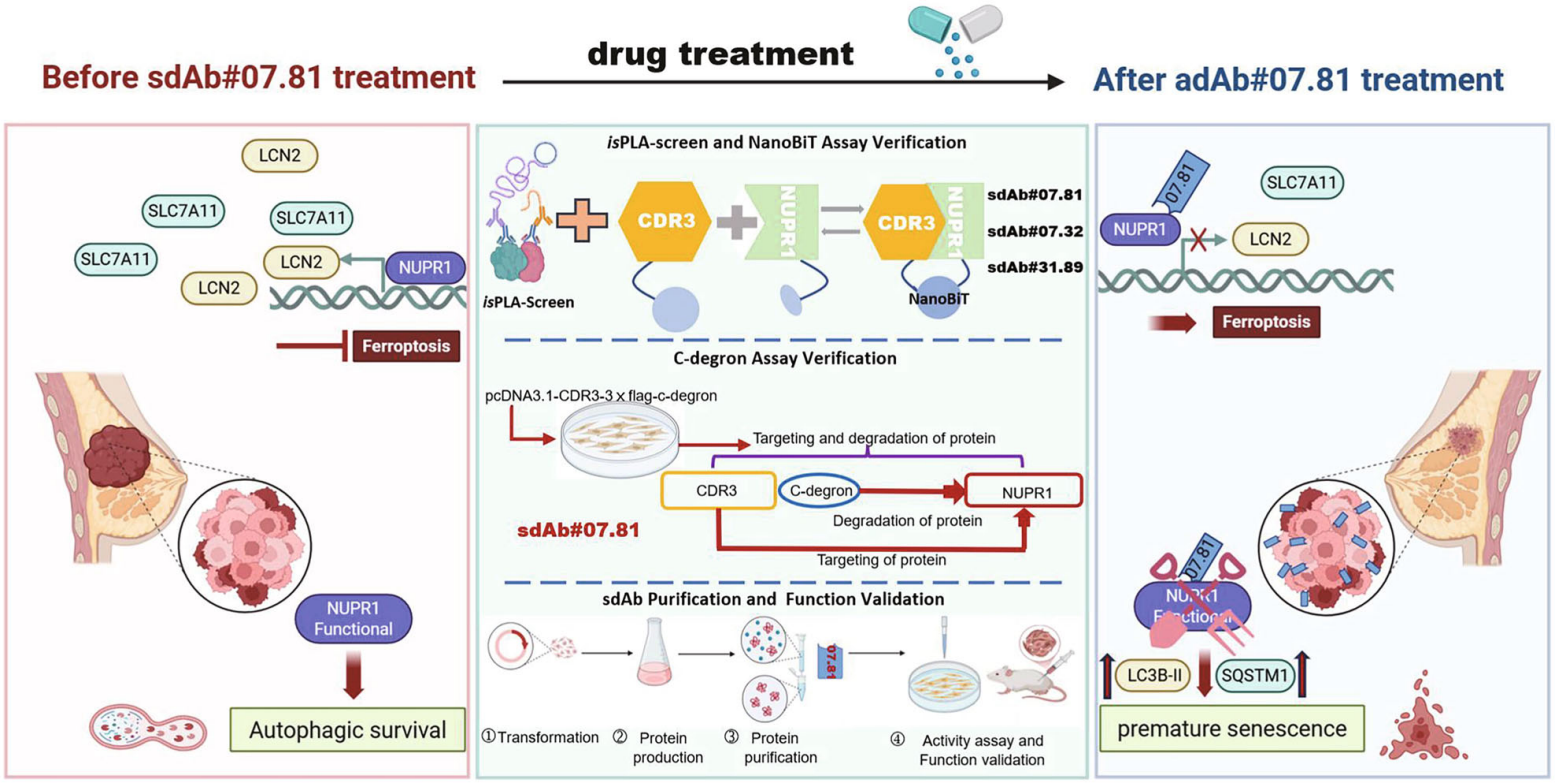
A working model. A high-throughput screening platform includes *is*PLA screen, NanoBiT assay, C-degron assay, purification, and functional validation in vitro and in vivo. Using this platform, we demonstrate that sdAb#07.81 degrades NUPR1, thereby derepressing ferroptosis and inducing premature senescence, which reveals its therapeutic potential for triple-negative breast cancer (TNBC).

Taken together, this study validates a high-throughput screening platform and identifies anti-NUPR1 sdAb#07.81 as a promising therapeutic candidate that suppresses TNBC progression in vitro and in vivo by mediating NUPR1 degradation to activate ferroptosis and premature senescence (Fig. 7).

## Discussion

This study successfully establishes a robust, high-throughput platform for screening sdAbs targeting NUPR1 and highlights the therapeutic potential of sdAb#07.81 in TNBC, presenting a novel therapeutic avenue that warrants further investigation.

NUPR1, a stress-inducible protein, is critically involved in cellular stress adaptation mechanisms, including chromatin remodeling, autophagy, premature senescence, and ferroptosis [6–9, 39]. In TNBC, which lacks targeted therapies and relies primarily on chemotherapy, NUPR1 plays an integral role in tumor progression, therapy resistance, and poor clinical outcomes [41]. Immunohistochemistry analysis in this study confirmed elevated NUPR1 expression in TNBC tissues, which was associated with poor prognosis. These findings corroborate previous studies highlighting NUPR1 as a crucial mediator of cancer cell survival under hostile microenvironments, such as hypoxia [42] and nutrient deprivation [43]. Currently, clinical trials for the specific NUPR1 small-molecule inhibitor ZZW-115 are actively ongoing [14, 37]. The NUPR1 inhibitor ZZW-115 could block stress granule (SG) formation, induce apoptosis, and reduce pancreatic intraepithelial neoplasia development in Kras^G12D^-driven tumors, highlighting its potential as a therapeutic strategy for pancreatic cancer [38, 39, 44] and other cancers [45, 46].

Targeting NUPR1 thus represents an innovative strategy to overcome therapeutic resistance and improve outcomes in TNBC patients. The high-throughput screening platform developed in this study demonstrated exceptional efficacy in identifying functional sdAbs targeting NUPR1. By integrating *is*PLA-seq [34] for initial CDR3 sequence identification, NanoBiT assays for specific binding confirmation, and C-degron degradation assays for functional validation, the platform efficiently narrowed down candidates from a large library to a few promising sdAbs. The identified sdAb#07.81 exhibited superior binding affinity and functional activity, as validated across multiple TNBC cell lines. These results highlight the utility of the screening strategy in addressing challenging intracellular targets like NUPR1, which are often inaccessible to conventional monoclonal antibodies [47, 48]. The functional experiments, including live-cell imaging, immunoprecipitation, and SPR assays, further validated the interaction between sdAb#07.81 and NUPR1, demonstrating its ability to directly bind and induce degradation of endogenous NUPR1. Importantly, in vitro studies showed that sdAb#07.81 not only reduced NUPR1 protein levels but also significantly inhibited TNBC cell growth, supporting its functional relevance. Mechanistically, both RNA sequencing analysis and GLB1/C11-BODIPY staining demonstrated that sdAb#07.81 treatment potently activated pathways associated with ferroptosis and premature senescence, using ZZW-115 as a reference. These findings were consistently reproducible across multiple human TNBC cell lines. Therefore, sdAb#07.81 likely suppresses TNBC progression through a concerted mechanism involving the derepression of ferroptosis and the induction of premature senescence. Concurrently, it also significantly downregulated DNA repair, mTORC1 signaling, MYC targets, cell cycle, and other cancer-related pathways. These findings affirm the efficacy of the screening platform in identifying therapeutically relevant sdAbs and provide a foundation for its application to other intracellular targets.

Single-domain antibodies, derived from camelid heavy-chain-only antibodies, offer several advantages over conventional therapeutic antibodies, including smaller size, higher stability, and the ability to penetrate tumors and target intracellular epitopes [28, 47]. The development of sdAb#07.81, as demonstrated in this study, represents a significant step forward in exploiting these advantages for clinical applications [49, 50]. In vivo experiments using a 4T1 cell-derived allograft immunocompetent BALB/c murine model provided compelling evidence for the therapeutic potential of sdAb#07.81. Treatment with sdAb#07.81 significantly reduced tumor size and weight and suppressed cell proliferation and exhibited no significant toxic side effects. These results establish sdAb#07.81 as a promising therapeutic candidate for TNBC and emphasize the translational potential of the developed platform. Given the challenges associated with TNBC, including its aggressive nature and lack of effective targeted therapies, the development of anti-NUPR1 sdAbs could address a critical unmet clinical need.

While this study provides robust evidence for the therapeutic potential of sdAb#07.81, further investigation is needed to translate these findings into clinical applications. Detailed preclinical studies, including pharmacokinetics, toxicity, and immunogenicity assessments [51, 52], are essential to ensure the safety and efficacy of sdAb#07.81 in human patients. Additional preclinical evaluations will be performed in TNBC organoid, PDX (patient-derived xenograft), and MMTV-PyMT models to assess the mechanism of efficacy and safety profiles, benchmarked against ZZW-115. Additionally, optimizing the delivery mechanisms for sdAb-based therapeutics, such as nanoparticle encapsulation [53] or fusion with cell-penetrating peptides [54], could enhance their clinical utility. Future studies should also explore the combinatorial potential of sdAb#07.81 with existing therapies, such as chemotherapy or immune checkpoint inhibitors, like carboplatin or anti-PD-1, to evaluate synergistic effects and broaden its therapeutic applications. Moreover, extending the application of the developed platform to identify sdAbs targeting other intracellular proteins could pave the way for novel therapeutic strategies to treat a wide range of diseases, including cancer.

In summary, this study demonstrates the effectiveness of a high-throughput screening platform for discovering functional sdAbs and establishes the therapeutic potential of sdAb#07.81 in targeting NUPR1 in TNBC. By addressing a critical intracellular protein involved in tumor progression and therapy resistance, this work highlights the importance of developing sdAbs for clinical applications. Future research aimed at advancing sdAb#07.81 into clinical trials could provide a transformative treatment option for TNBC patients and set a precedent for the broader application of sdAbs in cancer therapy.

## Materials and methods

### Cell culture

Human embryonic kidney (HEK293F and HEK293T), human breast cancer (MDA-MB-231, MDA-MB-468, T47D, MCF-7) cell lines were cultured in DMEM supplemented with 10% FBS (SINSAGE TECH, South America), 2 mM Glutamine, and 100 units/mL penicillin and 0.1 mg/mL streptomycin. Mouse breast cancer (4T1) cell line and human breast cancer (BT549) cell line were cultured in RPMI 1640 supplemented with 10% FBS (SINSAGE TECH, South America), 2 mM Glutamine, and 100 units/mL penicillin and 0.1 mg/mL streptomycin. All cells were maintained at 37 °C and 1% O_2_, 5% CO_2_, and passaged using 0.25% trypsin/0.02% EDTA for dissociation at 80% confluence. All cell lines were acquired from the American Type Culture Collection (ATCC, Manassas, VA, USA).

### In vitro allograft tumor growth experiments

4T1 cell suspensions were counted and resuspended in PBS at a concentration of 1 × 10^6^ cells/mL and injected into the unilateral posterior flank of 17–20 g female BALB/c mice (Charles River Laboratories). We randomly divided these 21 mice into three groups, with 7 mice in each group. They were administered PBS, sdAb-Con, and sdAb#07.81, respectively. The tumor was injected every 3 days with purified sdAb (15 mg/kg). After 17 days, the tumors were removed. The tumors were photographed and then weight and volume of the tumors were measured. For further studies, the pieces were lysed in the corresponding extraction buffer. The two pieces from each group were used for WB. Other tumor tissues were stored in liquid nitrogen or fixed in 4% paraformaldehyde used for IHC.

### *is*PLA and *is*PLA-seq

#### *Is*PLA-seq

Single-domain antibody library screening against NUPR1 by *is*PLA-seq in HEK293T cells was performed according to a previous approach [34, 55]. First, the Flag-tagged sdAb cDNA library was co-transfected with the HA-tagged NUPR1 construct into HEK293T cells. After 48 h, the trypsinized cells were fixed with 1% paraformaldehyde followed by *is*PLA using the Duolink In Situ Red Starter Kit Mouse/Rabbit according to the manufacturer’s instructions (DUO92101; Sigma-Aldrich). After *is*PLA, HEK293T cells were washed twice with PBS buffer containing 1% BSA, 2 mM EDTA, and 0.1% NaN3, directly sorted on a BD FACS Aria II flow cytometer, and collected as PCR templates for CDR3 fragments amplification followed by DNA sequencing. CDR3 primer (5’-AGTAACTTGAGTACCTTGACC-3’) was used for CDR3 amplification.

#### *Is*PLA

*Is*PLA was performed using the Duolink In Situ Red Starter Kit Mouse/Rabbit according to the manufacturer’s instructions as previously described [34, 56]. Briefly, the collected cells were permeabilized in PBS containing 0.5% Triton-X-100 for 10 min. Samples were incubated with blocking solution for 1 h at 37 °C in a 1.5 ml tube and then 60 min at 37 °C with an anti-NUPR1 rabbit monoclonal antibody and anti-Flag mouse monoclonal antibody. Cells were then incubated for 60 min at 37 °C with a mix of the MINUS (anti-mouse) and PLUS (anti-rabbit) PLA probes. Hybridized probes were ligated using the Ligation-Ligase solution for 30 min at 37 °C and then amplified using the Amplification-Polymerase solution for 100 min at 37 °C.

#### Generation knockout cell lines

For CRISPR/Cas9-mediated knockout of *NUPR1* in MDA-MB-231 and BT549 cells, oligos targeting exon 2 (forward 1: 5’-CACCGTCTCGTGCCCCGCCAGGGCTG-3’; reverse 1: 5’-AAACCAGCCCTGGCGGGGCACGAGAC-3’; forward 2: 5’-CACCGCGTGCCCGCCAGGGCTGGGG-3’; reverse 2: 5’-AAACCCCCAGCCCTGGCGGGCACGC-3’; forward 3: 5’-CACCGAACACCAACCGCCCCAGCCC-3’; reverse 3: 5’-AAACGGGCTGGGGCGGTTGGTGTTC-3’; forward 4: 5’-CACCGACAGGAGGTGGAGGCCGGAA-3’; reverse 4: 5’-AAACTTCCGGCCTCCACCTCCTGTC-3’) were annealed and cloned into the lentiCRISPRv2 (Addgene #52961) vector.

For shRNA-mediated knockdown of LCN2 in MDA-MB-231 cells, human LCN2-shRNA sequence (forward: 5’-CCGGGTACTTCAAGATCACCCTCTACTCGA GTAGAGGGTGATCTTGAAGTACTTTTTG-3’; reverse: 5’-AATTCAAAAAGT ACTTCAAGATCACCCTCTACTCGAGTAGAGGGTGATCTTGAAGTAC-3’) were annealed and cloned into the shRNA vector.

To produce lentivirus, lentiCRISPRv2-NUPR1 plasmid or shRNA plasmid were co-transfected into HEK293T cells with the packaging plasmids pMD2.G (AddGene #12259) and psPAX2 (AddGene #12260) using PEI. Lentivirus particles were collected from the supernatants of HEK293T cells 60 h post-transfection and infected MDA-MB-231 and BT549 cells two times. And then cells were selected with puromycin.

#### TMA IHC analysis

The triple-negative breast cancer tissue microarray (HBreD-180Bc01) was purchased from Shanghai Outdo Biotech Co, Ltd (Shanghai, China). The triple-negative breast cancer patient tissue sections were immunohistochemically stained by the anti-NUPR1-specific antibody. Criteria for immunohistochemical staining were classified into four groups based on the staining level of these four antibodies: low staining marked as 0, 0.5^+^, 1^+^, and 1.5^+^, moderate staining as 2^+^ and 2.5^+^, strong staining as 3^+^ and 3.5^+^, and very strong staining as 4^+^.

#### NanoBiT assay

The DNA fragment of sdAb was inserted into the LgBiT vector, and the DNA fragment of NUPR1 was inserted into the SmBiT vector by homologous recombination. HEK293T cells were transiently co-transfected with the plasmid. Cells were harvested and assayed for luciferase activity using NanoBiT^®^ Protein:Protein Interaction System (Promega, N2014, USA) following the manufacturer’s instructions at 36 h after transfection.

#### Western blotting

Tissues or cell lysates were treated using RIPA lysis buffer containing 1× cocktail inhibitor (Biosharp; Cat#BL629B). Samples supplemented with 5× loading buffer were heated at 100 °C for 10 min and separated by SDS-PAGE. PVDF membranes were immunoblotted with primary antibodies overnight at 4 °C. After washing three times in TBST buffer, the membranes were incubated with HRP-conjugated secondary antibody for 1 hour at 4 °C. Enhanced chemiluminescence (#36208ES, YeaSen) was used to detect immobilized antibodies. Primary antibodies were used at the indicated concentrations for western blotting: mouse anti-β-Actin (#ab8226; Abcam, 1:2000), rabbit anti-NUPR1(purification in own laboratory, 1:1000), mouse anti-SQSTM1 (#ab56416; Abcam, 1:1000), rabbit anti-SLC7A11 (26864-1-AP; Proteintech, 1:1000), rabbit anti-Cyclin D1 (26939-1-AP; Proteintech, 1:1000), rabbit anti-LC3B (L7543; Sigma, 1:1000), rabbit anti-p21 (10883-1-AP; Proteintech, 1:500), rabbit anti-LCN2 (26991-1-AP; Proteintech, 1:1000), mouse anti-Lamin B1 (HX1846; Huaxingbio, 1:1000). Secondary antibodies included goat anti-mouse horseradish peroxidase (HRP) and goat anti-rabbit HRP (ABclonal, 1:5000). And the original Western blots images are included in the Supplemental Material.

#### Immunoprecipitation (IP)

Immunoprecipitation was performed according to a previously published approach. Briefly, HEK293T cells were transfected with Flag-tagged sdAbs. After 48 h, cells were collected in lysis buffer ((1× PBS, pH 7.4, 0.5% sodium deoxycholate, 1% Triton-X-100, and 0.1% SDS) followed by centrifugation at 12,000 rpm for 15 min. Anti-Flag M2 Affinity gel (Cat#20584ES; YeaSen) was added to the supernatants at 4 °C overnight. After the incubation, beads were washed four times with ice-cold PIPA buffer, the beads were heated in 50 μl of 2×loading buffer at 100 °C for 10 min for western blotting.

#### GST pull-down assay

NUPR1 was cloned into pGEX-4T vector for expression with an N-terminal GST tag. GST and GST-NUPR1 proteins were expressed at 16 °C in BL21(DE3) cells. For GST pull-downs, BL21(DE3) cells were lysed by sonication in PBS. After centrifugation, the supernatant was purified on Glutathione Sepharose 4B beads in PBS. Purified GST or GST fusion proteins were immobilized on Glutathione Sepharose 4B beads. The beads were further incubated with purified Single-domain antibody proteins at 4 °C overnight and washed three times with ice-cold PBS. The beads were heated in 50 μl of 2×loading buffer at 100 °C for 10 min and were stained with Coomassie Brilliant Blue.

#### Immunofluorescence staining and confocal microscopy

4T1 cells were cultured on coverslips, washed with PBS three times, fixed in 4% formaldehyde for 15 min, permeabilized with 0.5%Triton-X-100 for 10 min, blocked with 5% bovine serum albumin for 30 mi,n and stained with specific primary antibodies overnight at 4 °C, followed by incubation with secondary antibody conjugated with Alexa Fluor 488 or 568 (Invitrogen) for 45 min at room temperature. Cells were also stained with 4’, 6-diamidino-2-phenylindole (DAPI) to visualize nuclei. Primary antibodies mouse anti-Flag (Cat#F1804; Sigma-Aldrich, 1:200), rabbit anti-NUPR1 (purification in own laboratory, 1:100) were used. The stained cells were visualized at 63× using confocal microscopy (Zeiss LSM 900 with Airyscan), and the image collecting software was Zen Black (ZEISS).

#### CCK-8 assay

Cells were seeded in 96-well plates with a fixed number of cells (4T1, 800 cells; MDA-MB-231, 1500 cells; BT549, 1500 cells) per well in triplicate. From the second day, cells were treated with CCK-8 reagent (YeaSen, #40203ES) at 37 °C for 2 h. And then, the absorbance at 450 nm was measured in a microplate reader (Synergy Neo2), and the growth curves were generated. All relevant assays were performed independently at least three times.

#### GLB1 staining

The cells were stained after fixation in 4% formaldehyde for 10 min with freshly prepared SA-β-galactosidase (SA-β-gal) staining solution overnight at 37 °C. GLB1 staining was performed using a Senescence β-Galactosidase Staining Kit (Solarbio, #G1580, Beijing, China) according to the manufacturer’s protocol. The number of GLB1-positive cells in randomly selected fields was expressed as a percentage of all cells counted. Cells were photographed under an automated live-cell fluorescence imaging system (Invitrogen EVOS M7000).

#### Lipid peroxidation assays

In total, 100,000 cells per well were plated in confocal 24-well plates 24 h before treatment. Following 24 to 48 h sdAb#07.81 or RSL3 treatment, cells were incubated with 5 µM C11-BODIPY 581/591 lipid peroxidation dye (Invitrogen D3861) for 1 h at 37 °C. Live-cell confocal imaging was then performed.

#### Immunohistochemistry

Tissues (mice or the triple-negative breast cancer tissue microarray-HBreD-180Bc01) were formalin-fixed and paraffin-embedded and then sectioned (5 μm). Sections were deparaffinized with xylene and rehydrated with ethanol. Then, 3% hydrogen peroxide was used to eliminate endogenous peroxidase 15 min at room temperature, followed by antigen retrieval in sodium citrate, pH 6.0, for 30 min in a 100 °C-water bath. The specimens were then incubated with primary antibodies at 4 °C overnight. The PV9000 2-step plus Poly-HP anti-mouse/rabbit IgG detection system and PV6001 2-step (rabbit), PV6002 2-step (mouse), or PV9001 2-step (human) plus Poly-HP anti-rabbit/goat IgG detection system (ZSGB-Bio) was applied. Diaminobenzidine (DAB) was used as a substrate (ChemMate Detection Kit, DAKO, Glostrup, Denmark), and hematoxylin was used as a counterstain. Immunostaining was examined and photographed with a whole-slide imaging system (Olympus VS200).

#### Transcriptome sequencing (RNA-Seq)

To identify NUPR1-regulated genes, 4T1 cells were transfected with purified Single-domain antibody proteins. The total RNAs were isolated using the Trizol reagent (Invitrogen) following the manufacturer’s protocol. RNA quality was inspected using Nanodrop (all samples had 260/280-ratio≥1.9). Four groups (Con group, sdAb#07.81 (24 h) group, sdAb-Con (48 h), sdAb#07.81 (48 h) group) were sequenced on an Illumina NovaSeq 6000 sequencing platform. Genes with a false discovery rate (FDR) < 0.05 were considered to be differentially expressed. Pathway analysis (KEGG, Kyoto Encyclopedia of Genes and Genomes) was performed with Annotation. Gene Ontology (GO) enrichment analysis was performed with GENE ontology unifying biology (http://geneontology.org/docs/go-enrichmentanalysis/) using all related genes (*P* < 0.05).

#### Recombinant protein expression and purification

For recombinant protein expression in HEK293F cells, a plasmid encoding a secretion signal peptide, the CDR3 sequence of NUPR1, a Tat transmembrane domain, a 3×Flag tag, and a His tag was constructed. All the constructs were verified by DNA sequencing. The recombinant protein purification assay was performed as described. Briefly, HEK293F cells transfected by the expression vector were cultured in Union medium (Union, Shanghai) at 37 °C. After 72 h, the cells were centrifuged for 20 min at 4000×*g* and 4 °C. The cell debris was removed by centrifugation. After adding PMSF to the supernatant and through a 0.45-μm filter, the supernatant was loaded onto Ni²⁺-NTA affinity column (C600793, Sangon Biotech, Shanghai) for protein purification and washed with 500 mM imidazole. After washing, the eluted samples were analyzed by using SDS-PAGE and stained with Coomassie Brilliant Blue.

#### Statistical analysis

All values and error bars in graphs are means ± standard error of the mean (means ± SD). Comparisons between two groups were made using two-tailed Student’s *t* test. Statistical significance was considered for *P < 0.05, **P < 0.01, ***P < 0.001, ****P < 0.0001, and ns = not significant. Analyses were carried out using GraphPad Prism 9.0 (GraphPad Software Inc., La Jolla, CA).

## Supplementary information

Supplementary Figure

Supplementary Original Western Blots

## Data Availability

The raw RNA-seq data used in this study are available in the Sequence Read Archive. The raw data and processed data of RNA-seq data generated in this study have been deposited in the Gene Expression Omnibus (GEO) database under accession code GSE300149. All the other data supporting the findings of this study are available within the article and its Supplementary Information files.

## References

[1] Zhu S, Wu Y, Song B, Yi M, Yan Y, Mei Q, et al. Recent advances in targeted strategies for triple-negative breast cancer. J Hematol Oncol. 2023;16:100. 37641116 10.1186/s13045-023-01497-3 PMC10464091

[2] Guo J, Hu J, Zheng Y, Zhao S, Ma J. Artificial intelligence: opportunities and challenges in the clinical applications of triple-negative breast cancer. Br J Cancer. 2023;128:2141–9. 36871044 10.1038/s41416-023-02215-z PMC10241896

[3] Li Y, Zhang H, Merkher Y, Chen L, Liu N, Leonov S, et al. Recent advances in therapeutic strategies for triple-negative breast cancer. J Hematol Oncol. 2022;15:121. 36038913 10.1186/s13045-022-01341-0 PMC9422136

[4] Liu Y, Hu Y, Xue J, Li J, Yi J, Bu J, et al. Advances in immunotherapy for triple-negative breast cancer. Mol cancer. 2023;22:145. 37660039 10.1186/s12943-023-01850-7 PMC10474743

[5] de Paula B, Kieran R, Koh SSY, Crocamo S, Abdelhay E, Muñoz-Espín D. Targeting senescence as a therapeutic opportunity for triple-negative breast cancer. Mol Cancer Ther. 2023;22:583–98. 36752780 10.1158/1535-7163.MCT-22-0643 PMC10157365

[6] Liu S, Costa M. The role of NUPR1 in response to stress and cancer development. Toxicol Appl Pharmacol. 2022;454:116244. 36116561 10.1016/j.taap.2022.116244

[7] Martin TA, Li AX, Sanders AJ, Ye L, Frewer K, Hargest R, et al. NUPR1 and its potential role in cancer and pathological conditions (review). Int J Oncol. 2021;58:21. 33760183 10.3892/ijo.2021.5201

[8] Huang C, Santofimia-Castaño P, Iovanna J. NUPR1: a critical regulator of the antioxidant system. Cancers. 2021;13:3670. 34359572 10.3390/cancers13153670 PMC8345110

[9] Cano CE, Hamidi T, Sandi MJ, Iovanna JL. Nupr1: the Swiss-knife of cancer. J Cell Physiol. 2011;226:1439–43. 20658514 10.1002/jcp.22324

[10] Neira JL, Rizzuti B, Jiménez-Alesanco A, Abián O, Velázquez-Campoy A, Iovanna JL. The paralogue of the intrinsically disordered nuclear protein 1 has a nuclear localization sequence that binds to human importin α3. Int J Mol Sci. 2020;21:7428. 33050086 10.3390/ijms21197428 PMC7583046

[11] Jin HO, Seo SK, Woo SH, Choe TB, Hong SI, Kim JI, et al. Nuclear protein 1 induced by ATF4 in response to various stressors acts as a positive regulator on the transcriptional activation of ATF4. IUBMB Life. 2009;61:1153–8. 19946894 10.1002/iub.271

[12] Fan T, Wang X, Zhang S, Deng P, Jiang Y, Liang Y, et al. NUPR1 promotes the proliferation and metastasis of oral squamous cell carcinoma cells by activating TFE3-dependent autophagy. Signal Transduct Target Ther. 2022;7:130. 35462576 10.1038/s41392-022-00939-7 PMC9035452

[13] Wang L, Sun J, Yin Y, Sun Y, Ma J, Zhou R, et al. Transcriptional coregualtor NUPR1 maintains tamoxifen resistance in breast cancer cells. Cell Death Dis. 2021;12:149. 33542201 10.1038/s41419-021-03442-z PMC7862277

[14] Huang C, Santofimia-Castaño P, Liu X, Xia Y, Peng L, Gotorbe C, et al. NUPR1 inhibitor ZZW-115 induces ferroptosis in a mitochondria-dependent manner. Cell Death Discov. 2021;7:269. 34599149 10.1038/s41420-021-00662-2 PMC8486797

[15] Liu J, Song X, Kuang F, Zhang Q, Xie Y, Kang R, et al. NUPR1 is a critical repressor of ferroptosis. Nat Commun. 2021;12:647. 33510144 10.1038/s41467-021-20904-2 PMC7843652

[16] Santofimia-Castaño P, Xia Y, Peng L, Velázquez-Campoy A, Abián O, Lan W, et al. Targeting the stress-induced protein NUPR1 to treat pancreatic adenocarcinoma. Cells. 2019;8:1453. 31744261 10.3390/cells8111453 PMC6912534

[17] Mu Y, Yan X, Li D, Zhao D, Wang L, Wang X, et al. NUPR1 maintains autolysosomal efflux by activating SNAP25 transcription in cancer cells. Autophagy. 2018;14:654–70. 29130426 10.1080/15548627.2017.1338556 PMC5959327

[18] Su SB, Motoo Y, Iovanna JL, Xie MJ, Mouri H, Ohtsubo K, et al. Expression of p8 in human pancreatic cancer. Clin Cancer Res. 2001;7:309–13. 11234885

[19] Malicet C, Lesavre N, Vasseur S, Iovanna JL. p8 inhibits the growth of human pancreatic cancer cells and its expression is induced through pathways involved in growth inhibition and repressed by factors promoting cell growth. Mol Cancer. 2003;2:37. 14613582 10.1186/1476-4598-2-37 PMC280693

[20] Bingham C, Dickinson D, Cray J, Koli K, Ogbureke KU. Expression of p8 in human oral squamous cell carcinoma. Head Neck Pathol. 2015;9:214–22. 25155047 10.1007/s12105-014-0565-1 PMC4424217

[21] Wang L, Wen J, Sun Y, Yang X, Ma Y, Tian X. Knockdown of NUPR1 inhibits angiogenesis in lung cancer through IRE1/XBP1 and PERK/eIF2α/ATF4 signaling pathways. Open Med. 2023;18:20230796. 10.1515/med-2023-0796 PMC10579879 37854285

[22] Lee YK, Jee BA, Kwon SM, Yoon YS, Xu WG, Wang HJ, et al. Identification of a mitochondrial defect gene signature reveals NUPR1 as a key regulator of liver cancer progression. Hepatology. 2015;62:1174–89. 26173068 10.1002/hep.27976 PMC6312643

[23] Chen CY, Wu SM, Lin YH, Chi HC, Lin SL, Yeh CT, et al. Induction of nuclear protein-1 by thyroid hormone enhances platelet-derived growth factor A mediated angiogenesis in liver cancer. Theranostics. 2019;9:2361–79. 31149049 10.7150/thno.29628 PMC6531305

[24] Lu Y, Chen W, Xuan Y, Li X, Wu S, Wang H, et al. ATF4/NUPR1 axis promotes cancer cell survival and mediates immunosuppression in clear cell renal cell carcinoma. Discov Oncol. 2024;15:607. 39480570 10.1007/s12672-024-01485-0 PMC11528094

[25] Tan M, He Y, Yi J, Chen J, Guo Q, Liao N, et al. WTAP mediates NUPR1 regulation of LCN2 through m(6)A modification to influence ferroptosis, thereby promoting breast cancer proliferation, migration and invasion. Biochem Genet. 2024;62:876–91. 37477758 10.1007/s10528-023-10423-8

[26] Ree AH, Pacheco MM, Tvermyr M, Fodstad O, Brentani MM. Expression of a novel factor, com1, in early tumor progression of breast cancer. Clin Cancer Res. 2000;6:1778–83. 10815897

[27] Wang Z, Zhi K, Ding Z, Sun Y, Li S, Li M, et al. Emergence in protein derived nanomedicine as anticancer therapeutics: more than a tour de force. Semin Cancer Biol. 2021;69:77–90. 31962173 10.1016/j.semcancer.2019.11.012

[28] Kunz S, Durandy M, Seguin L, Feral CC. NANOBODY(®) molecule, a giga medical tool in nanodimensions. Int J Mol Sci. 2023;24:13229. 37686035 10.3390/ijms241713229 PMC10487883

[29] Al-Baradie RS. Nanobodies as versatile tools: A focus on targeted tumor therapy, tumor imaging and diagnostics. Hum Antibodies. 2020;28:259–72. 32831197 10.3233/HAB-200425

[30] Yu X, Xu Q, Wu Y, Jiang H, Wei W, Zulipikaer A, et al. Nanobodies derived from Camelids represent versatile biomolecules for biomedical applications. Biomater Sci. 2020;8:3559–73. 32490444 10.1039/d0bm00574f

[31] Steeland S, Vandenbroucke RE, Libert C. Nanobodies as therapeutics: big opportunities for small antibodies. Drug Discov today. 2016;21:1076–113. 27080147 10.1016/j.drudis.2016.04.003

[32] Kang-Pettinger T, Walker K, Brown R, Cowan R, Wright H, Baravalle R, et al. Identification, binding, and structural characterization of single domain anti-PD-L1 antibodies inhibitory of immune regulatory proteins PD-1 and CD80. J Biol Chem. 2023;299:102769. 36470427 10.1016/j.jbc.2022.102769 PMC9811221

[33] Kuang Z, Pu P, Wu M, Wu Z, Wang L, Li Y, et al. A novel bispecific antibody with PD-L1-assisted OX40 activation for cancer treatment. Mol Cancer Ther. 2020;19:2564–74. 32999045 10.1158/1535-7163.MCT-20-0226

[34] Yin Y, Yan F, Zhou R, Li M, Ma J, Liu Z, et al. Single-domain antibody screening by isPLA-seq. Life Sci Alliance. 2022;5:e202101115. 34675071 10.26508/lsa.202101115 PMC8548206

[35] Takeuchi J, Chen H, Coffino P. Proteasome substrate degradation requires association plus extended peptide. EMBO J. 2006;26:123–31. 17170706 10.1038/sj.emboj.7601476 PMC1782366

[36] Lan W, Santofimia-Castaño P, Swayden M, Xia Y, Zhou Z, Audebert S, et al. ZZW-115–dependent inhibition of NUPR1 nuclear translocation sensitizes cancer cells to genotoxic agents. JCI Insight. 2020;5:e138117. 32780723 10.1172/jci.insight.138117 PMC7526551

[37] Lan W, Santofimia-Castaño P, Xia Y, Zhou Z, Huang C, Fraunhoffer N, et al. Targeting NUPR1 with the small compound ZZW-115 is an efficient strategy to treat hepatocellular carcinoma. Cancer Lett. 2020;486:8–17. 32446862 10.1016/j.canlet.2020.04.024

[38] Santofimia-Castaño P, Fraunhoffer N, Liu X, Bessone IF, di Magliano MP, Audebert S, et al. Targeting NUPR1-dependent stress granules formation to induce synthetic lethality in KrasG12D-driven tumors. EMBO Mol Med. 2024;16:475–505. 38360999 10.1038/s44321-024-00032-2 PMC10940650

[39] Zhuang X, Wang Q, Joost S, Ferrena A, Humphreys DT, Li Z, et al. Ageing limits stemness and tumorigenesis by reprogramming iron homeostasis. Nature. 2024;637:184–94. 39633048 10.1038/s41586-024-08285-0

[40] Lan W, Santofimia-Castaño P, Swayden M, Xia Y, Zhou Z, Audebert S, et al. ZZW-115-dependent inhibition of NUPR1 nuclear translocation sensitizes cancer cells to genotoxic agents. JCI Insight. 2020;5:e138117. 32780723 10.1172/jci.insight.138117 PMC7526551

[41] Ortiz A, Stavrou A, Liu S, Chen D, Shen SS, Jin C. NUPR1 packaged in extracellular vesicles promotes murine triple-negative breast cancer in a type 1 interferon-independent manner. Extracell Vesicles Circ Nucleic Acids. 2024;5:19–36. 10.20517/evcna.2023.59 PMC10887431 38405101

[42] Li T, Fu X, Wang J, Shang W, Wang X, Zhang L, et al. Mechanism of NURP1 in temozolomide resistance in hypoxia-treated glioma cells via the KDM3A/TFEB axis. Oncol Res. 2023;31:345–59. 37305393 10.32604/or.2023.028724 PMC10229302

[43] Hamidi T, Algül H, Cano CE, Sandi MJ, Molejon MI, Riemann M, et al. Nuclear protein 1 promotes pancreatic cancer development and protects cells from stress by inhibiting apoptosis. J Clin Investig. 2012;122:2092–103. 22565310 10.1172/JCI60144 PMC3366404

[44] Santofimia-Castaño P, Lan W, Estaras M, Xia Y, Cosialls E, Fraunhoffer N, et al. Mechanisms of induced resistance to the antitumoral agent ZZW-115 in pancreas ductal adenocarcinoma. Sci Rep. 2025;15:27242. 40715463 10.1038/s41598-025-11931-w PMC12297348

[45] Zhang X-Y, Li S-S, Gu Y-R, Xiao L-X, Ma X-Y, Chen X-R, et al. CircPIAS1 promotes hepatocellular carcinoma progression by inhibiting ferroptosis via the miR-455-3p/NUPR1/FTH1 axis. Mol cancer. 2024;23:113. 38802795 10.1186/s12943-024-02030-x PMC11131253

[46] Fang Y, Chen H, Liu Y, Jiang K, Qian Y, Wei J, et al. NUPR1 promotes radioresistance in colorectal cancer cells by inhibiting ferroptosis. J Cell Mol Med. 2025;29:e70519. 40176685 10.1111/jcmm.70519 PMC11965884

[47] Muyldermans S. Nanobodies: natural single-domain antibodies. Annu Rev Biochem. 2013;82:775–97. 23495938 10.1146/annurev-biochem-063011-092449

[48] Anderson GP, Liu JH, Zabetakis D, Liu JL, Goldman ER. Thermal stabilization of anti-α-cobratoxin single domain antibodies. Toxicon. 2017;129:68–73. 28209480 10.1016/j.toxicon.2017.02.008

[49] Chen T, Liu X, Hong H, Wei H. Novel single-domain antibodies against the EGFR domain III epitope exhibit the anti-tumor effect. J Transl Med. 2020;18:376. 33023595 10.1186/s12967-020-02538-y PMC7541222

[50] Iezzi ME, Policastro L, Werbajh S, Podhajcer O, Canziani GA. Single-domain antibodies and the promise of modular targeting in cancer imaging and treatment. Front Immunol. 2018;9:273. 29520274 10.3389/fimmu.2018.00273 PMC5827546

[51] Hejmady S, Pradhan R, Kumari S, Pandey M, Dubey SK, Taliyan R. Pharmacokinetics and toxicity considerations for antibody-drug conjugates: an overview. Bioanalysis. 2023;15:1193–202. 37724472 10.4155/bio-2023-0104

[52] Mahalingaiah PK, Ciurlionis R, Durbin KR, Yeager RL, Philip BK, Bawa B, et al. Potential mechanisms of target-independent uptake and toxicity of antibody-drug conjugates. Pharmacol Ther. 2019;200:110–25. 31028836 10.1016/j.pharmthera.2019.04.008

[53] Sánchez A, Mejía SP, Orozco J. Recent advances in polymeric nanoparticle-encapsulated drugs against intracellular infections. Molecules. 2020;25:3760. 32824757 10.3390/molecules25163760 PMC7464666

[54] Fei L, Yap LP, Conti PS, Shen WC, Zaro JL. Tumor targeting of a cell penetrating peptide by fusing with a pH-sensitive histidine-glutamate co-oligopeptide. Biomaterials. 2014;35:4082–7. 24508076 10.1016/j.biomaterials.2014.01.047 PMC3954778

[55] Ma J, Xu J, Gao Q, Sun Y, Wang Y, Liu Z, et al. Engineering single-domain antibodies targeting Gasdermin E activation by the chemotherapeutic agent cis-diaminodichloroplatinum. Biotechnol J. 2023;18:e2200633. 37204010 10.1002/biot.202200633

[56] Soderberg O, Gullberg M, Jarvius M, Ridderstrale K, Leuchowius KJ, Jarvius J, et al. Direct observation of individual endogenous protein complexes in situ by proximity ligation. Nat Methods. 2006;3:995–1000. 17072308 10.1038/nmeth947

