# Supplementary material for: Targeting intrinsically disordered nuclear protein 1 (NUPR1) with single-domain antibodies alleviates triple-negative breast cancer (TNBC) progression in vivo
Source: Cell Death Dis. 2025 Dec 22;16(1):913. doi: 10.1038/s41419-025-08332-2 (PMC12748981; doi:10.1038/s41419-025-08332-2)
Supplement: Supplementary file 1 — Supplementary Figure [file 41419_2025_8332_MOESM1_ESM.pdf]

Supplementary materials for

Targeting intrinsically disordered nuclear protein 1 (NUPR1) with single-domain antibodies alleviates triple-negative breast cancer (TNBC) progression *in vivo*

Tianzhuo Wang*[#1], Min Wang*[1], Xuanru Chen[1], Yueyuan Yin[3], Jintao Xu[2], Yanan Sun[2], Ailing Wu[1], Zhe Liu[1], Zhenyi Ma[#1]

Fig. S1

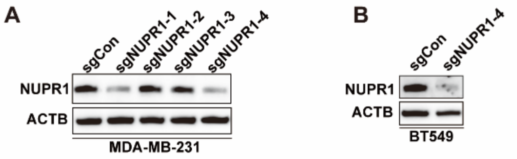

Fig S1. Establishment of *NUPR1*-knockout TNBC cell lines.

A) CRISPR-Cas9 mediated *NUPR1* knockout in MDA-MB-231 cells, and the knockout efficiency was identified by WB with anti-NUPR1 antibody. ACTB as a loading control.

B) CRISPR-Cas9 mediated NUPR1 knockout in BT549 cells, and the knockout efficiency was identified by WB with anti-NUPR1 antibody. ACTB as a loading control.

Fig. S2

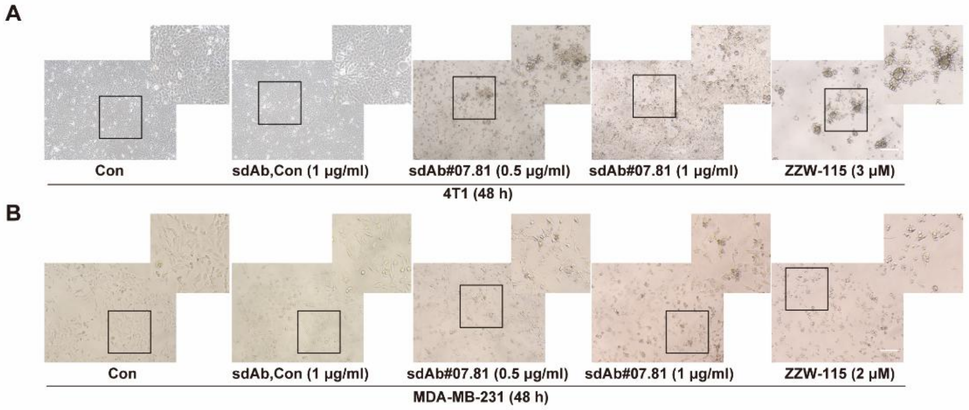

Fig S2. Functional validation of Flag-tagged anti-NUPR1 sdAb#07.81 *in vitro*.

A) Cell images of 4T1 cells were treated with purified anti-NUPR1 sdAb clone Con, sdAb#07.81 or ZZW-115 at the indicated concentration for 48 h, compared with Con. Scale bars, 100 µm.

B) Cell images of MDA-MB-231 cells were treated with purified anti-NUPR1 sdAb clone Con, sdAb#07.81 or ZZW-115 at the indicated concentration for 48 h, compared with Con. Scale bars, 100 µm.

Fig. S3

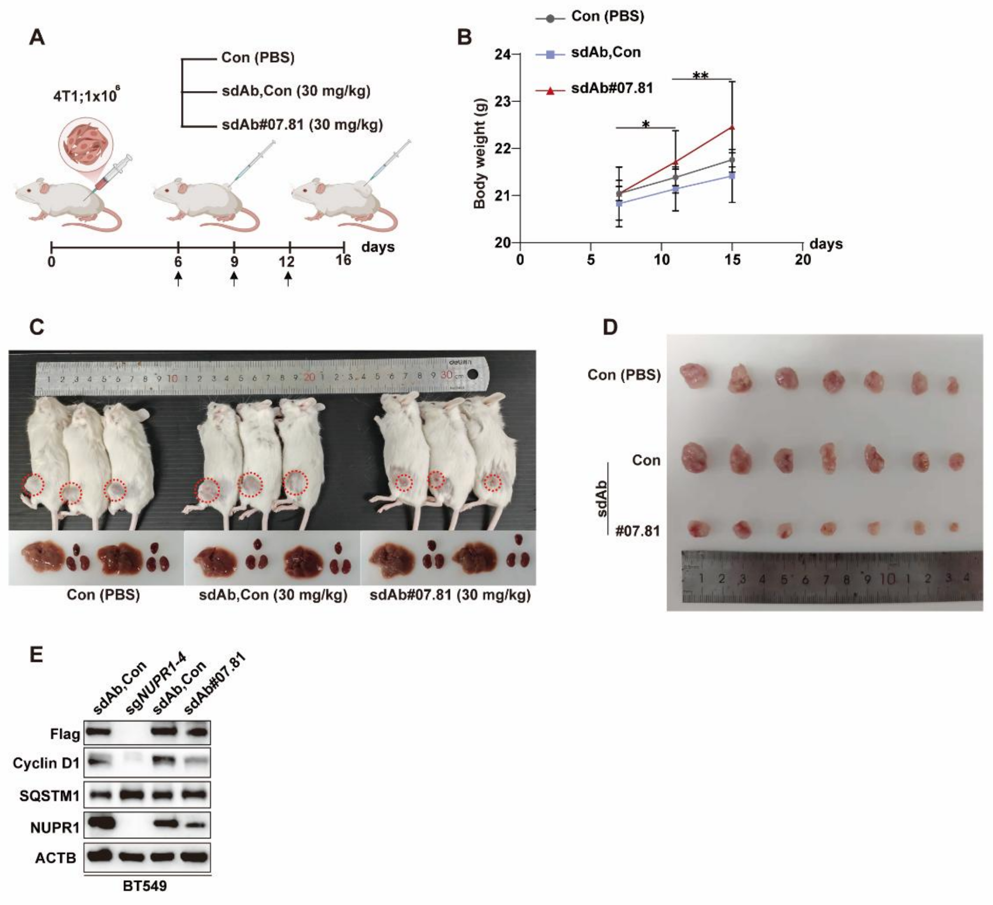

Fig S3. Anti-NUPR1 sdAb#07.81 inhibits 4T1 cell progression.

A) Allograft experiment was performed on BALB/c mice injected with 4T1 cells (1×10^6 cells per mouse). Groups of different mice that were Con ($n = 7$), sdAb-Con ($n = 7$), or sdAb#07.81 ($n = 7$). Tumor was injected every 3 days with purified sdAb proteins.

B) Groups of different mice weight were measured. Not significant (ns) by unpaired Student's *t* test. *$p < 0.05$, **$p < 0.01$ by unpaired Student's *t* test. Data are presented as the mean±SEM and are from one independent experiment with 7 mice per group.

C-D) Representative pictures of mice and main organs from Con, sdAb-Con and sdAb#07.81 treatment groups.

E) Immunoblot of Cyclin D1, SQSTM1, NUPR1 and Flag in BT549 cells treated with sgNUPR1, anti-NUPR1 sdAb-Con and sdAb#07.81(0.5 μg/mL) at the indicated concentration for 48 h, compared with Con. ACTB was used as a loading control.
