# Supplementary material for: Targeting intrinsically disordered nuclear protein 1 (NUPR1) with single-domain antibodies alleviates triple-negative breast cancer (TNBC) progression in vivo
Source: Cell Death Dis. 2025 Dec 22;16(1):913. doi: 10.1038/s41419-025-08332-2 (PMC12748981; doi:10.1038/s41419-025-08332-2)
Supplement: Supplementary file 2 — Supplementary Original Western Blots [file 41419_2025_8332_MOESM2_ESM.pdf]

Supplementary materials for

Targeting intrinsically disordered nuclear protein 1 (NUPR1) with single-domain antibodies alleviates triple-negative breast cancer (TNBC) progression *in vivo*

Tianzhuo Wang*#1, Min Wang*1, Xuanru Chen[1], Yueyuan Yin[3], Jintao Xu[2], Yanan Sun[2], Ailing Wu[1], Zhe Liu[1], Zhenyi Ma#1

Supplementary Original Western Blots

Supplemental information

Fig. 1

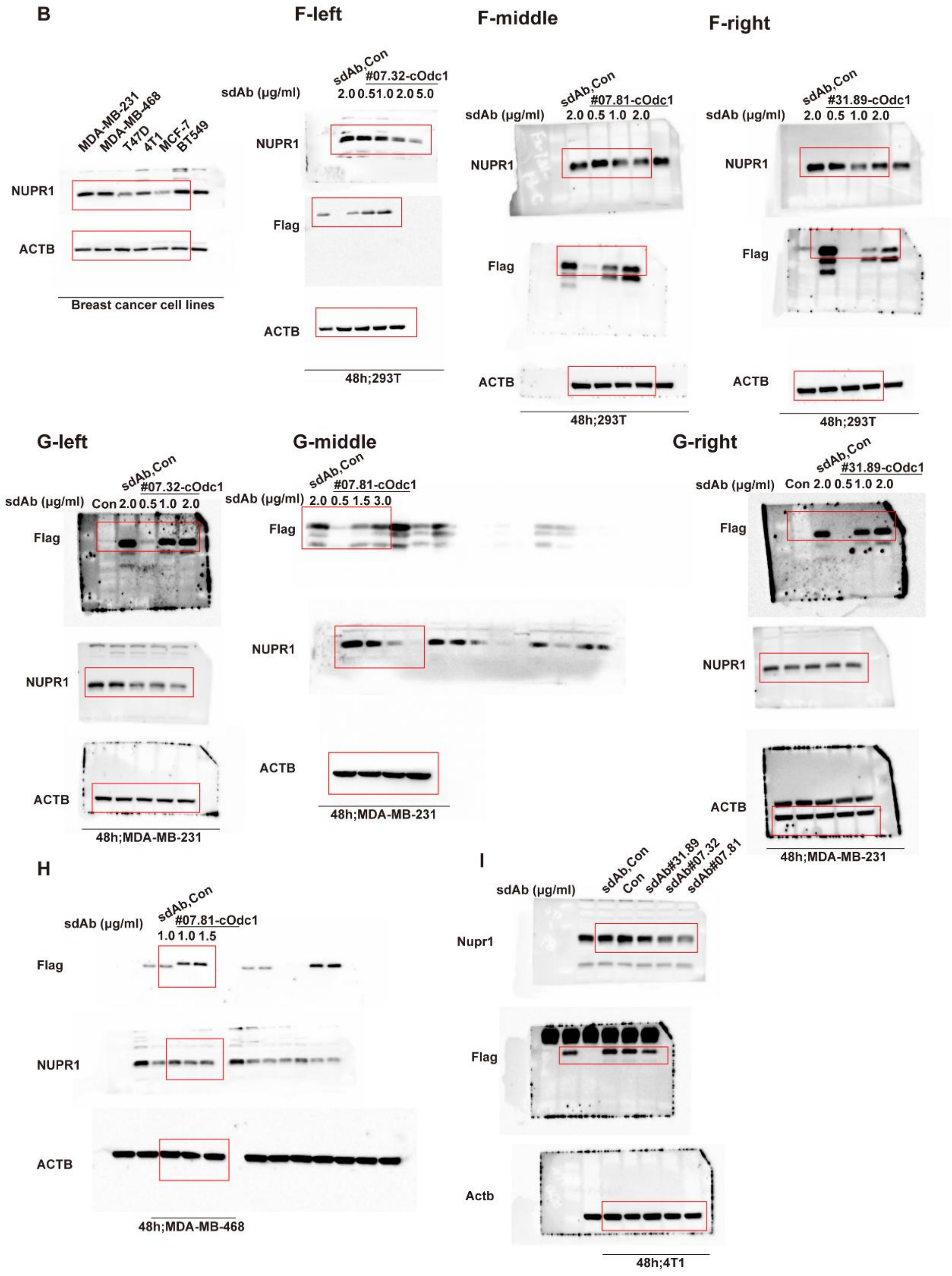

Fig. 2

D

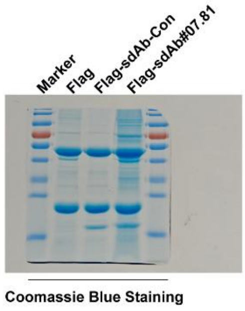
Marker
Flag
Flag-sdAb-Con
Flag-sdAb#07.81
Coomassie Blue Staining

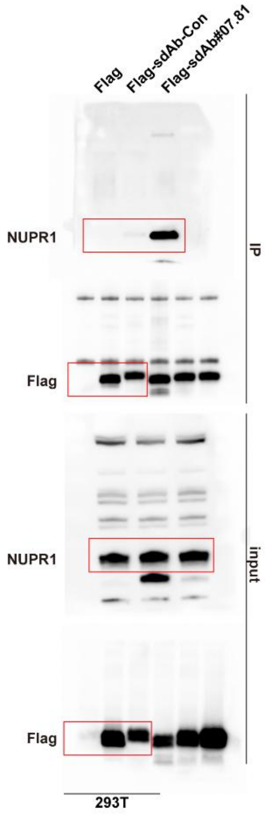
Flag
Flag-sdAb-Con
Flag-sdAb#07.81
NUPR1
Flag
IP
NUPR1
input
Flag
293T

Fig. 3

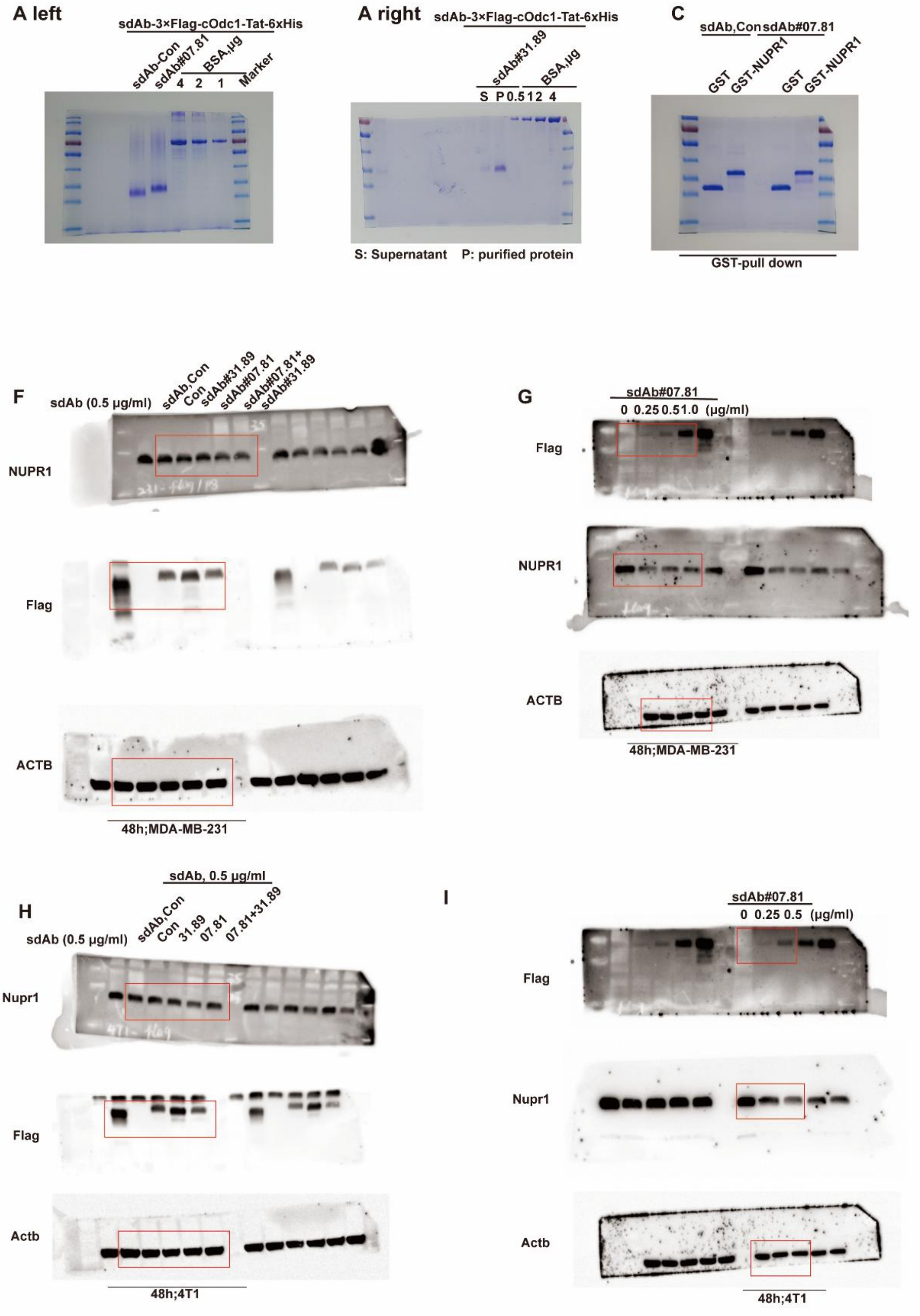
A left
sdAb-3×Flag-cOdc1-Tat-6xHis
sdAb-Con
sdAb#07.81
BSA,μg
4 2 1
Marker
A right
sdAb-3×Flag-cOdc1-Tat-6xHis
sdAb#31.89
BSA,μg
S P 0.5 1 2 4
S: Supernatant P: purified protein
C
sdAb,Con
sdAb#07.81
GST
GST-NUPR1
GST
GST-NUPR1
GST-pull down
F
sdAb (0.5 μg/ml)
sdAb,Con
Con
sdAb#31.89
sdAb#07.81
sdAb#07.81+ sdAb#31.89
NUPR1
Flag
ACTB
48h;MDA-MB-231
G
sdAb#07.81
0 0.25 0.5 1.0 (μg/ml)
Flag
NUPR1
ACTB
48h;MDA-MB-231
H
sdAb, 0.5 μg/ml
sdAb (0.5 μg/ml)
sdAb,Con
Con
31.89
07.81
07.81+31.89
Nupr1
Flag
Actb
48h;4T1
I
sdAb#07.81
0 0.25 0.5 (μg/ml)
Flag
Nupr1
Actb
48h;4T1

Fig. 4

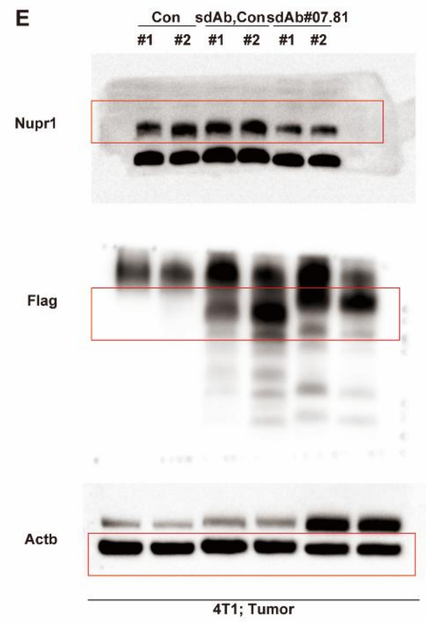
E
Con
sdAb,Con
sdAb#07.81
#1
#2
#1
#2
#1
#2
Nupr1
Flag
Actb
4T1; Tumor

Fig. 5

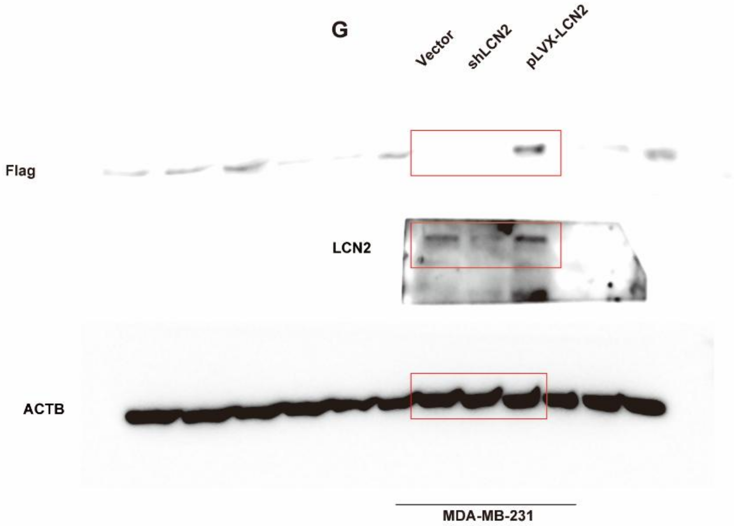
G
Vector
shLCN2
pLVX-LCN2
Flag
LCN2
ACTB
MDA-MB-231

Fig. 6

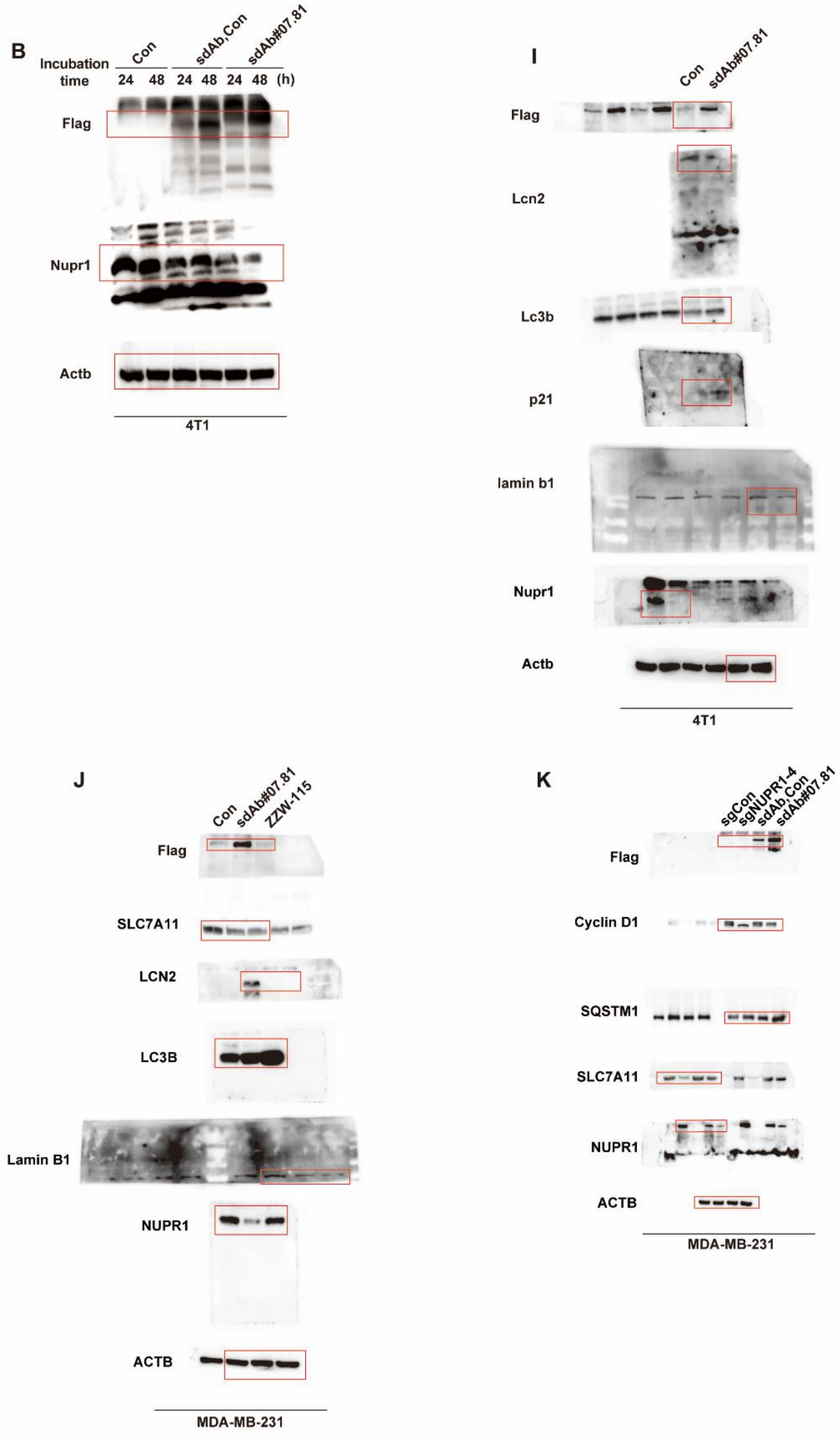

B
Incubation time
Con
sdAb.Con
sdAb#07.81
24 48 24 48 24 48 (h)
Flag
Nupr1
Actb
4T1
I
Con
sdAb#07.81
Flag
Lcn2
Lc3b
p21
lamin b1
Nupr1
Actb
4T1
J
Con
sdAb#07.81
ZZW-115
Flag
SLC7A11
LCN2
LC3B
Lamin B1
NUPR1
ACTB
MDA-MB-231
K
sgCon
sgNUPR1-4
sdAb.Con
sdAb#07.81
Flag
Cyclin D1
SQSTM1
SLC7A11
NUPR1
ACTB
MDA-MB-231

Fig. S1

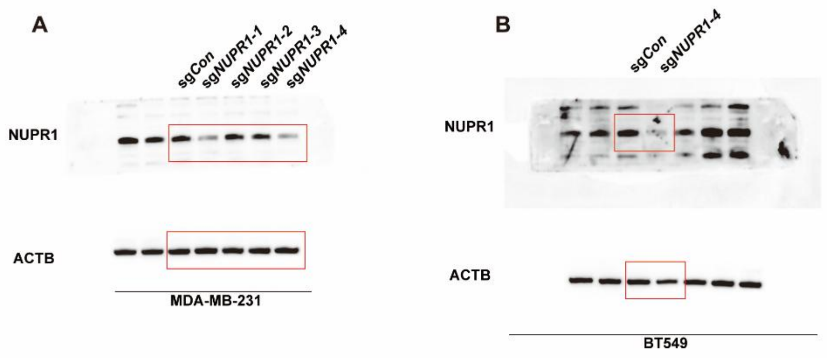
A
sgCon
sgNUPR1-1
sgNUPR1-2
sgNUPR1-3
sgNUPR1-4
NUPR1
ACTB
MDA-MB-231
B
sgCon
sgNUPR1-4
NUPR1
ACTB
BT549

Fig. S3

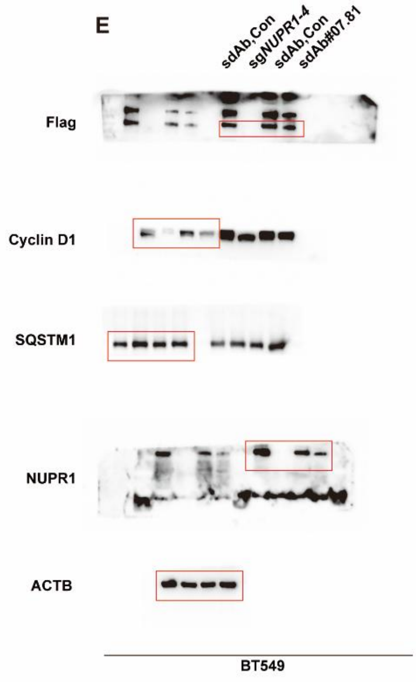
E
sdAb,Con
sgNUPR1-4
sdAb,Con
sdAb#07.81
Flag
Cyclin D1
SQSTM1
NUPR1
ACTB
BT549
